# Structural basis of DNA replication origin recognition by human Orc6 protein binding with DNA

**DOI:** 10.1093/nar/gkaa751

**Published:** 2020-09-28

**Authors:** Naining Xu, Yingying You, Changdong Liu, Maxim Balasov, Lee Tung Lun, Yanyan Geng, Chun Po Fung, Haitao Miao, Honglei Tian, To To Choy, Xiao Shi, Zhuming Fan, Bo Zhou, Katarina Akhmetova, Rahman Ud Din, Hongyu Yang, Quan Hao, Peiyuan Qian, Igor Chesnokov, Guang Zhu

**Affiliations:** Division of Life Science, The Hong Kong University of Science and Technology, Clear Water Bay, Kowloon, Hong Kong SAR, 00000, China; Department of Oral and Maxillofacial Surgery, Peking University ShenzhenHospital, Shenzhen Peking University-The Hong Kong University of Science and Technology Medical Center, Shenzhen, 518036, China; Division of Life Science, The Hong Kong University of Science and Technology, Clear Water Bay, Kowloon, Hong Kong SAR, 00000, China; Department of Oncology, Xiangya Hospital, Central South University, 87 Xiangya Road, Changsha, 410008, Hunan, China; Division of Life Science, The Hong Kong University of Science and Technology, Clear Water Bay, Kowloon, Hong Kong SAR, 00000, China; Department of Biochemistry and Molecular Genetics, University of Alabama at Birmingham School of Medicine, Birmingham, AL 35294, USA; Division of Life Science, The Hong Kong University of Science and Technology, Clear Water Bay, Kowloon, Hong Kong SAR, 00000, China; Division of Life Science, The Hong Kong University of Science and Technology, Clear Water Bay, Kowloon, Hong Kong SAR, 00000, China; Division of Life Science, The Hong Kong University of Science and Technology, Clear Water Bay, Kowloon, Hong Kong SAR, 00000, China; Division of Life Science, The Hong Kong University of Science and Technology, Clear Water Bay, Kowloon, Hong Kong SAR, 00000, China; Division of Life Science, The Hong Kong University of Science and Technology, Clear Water Bay, Kowloon, Hong Kong SAR, 00000, China; Division of Life Science, The Hong Kong University of Science and Technology, Clear Water Bay, Kowloon, Hong Kong SAR, 00000, China; Division of Life Science, The Hong Kong University of Science and Technology, Clear Water Bay, Kowloon, Hong Kong SAR, 00000, China; School of Biomedical Sciences, University of Hong Kong, 21 Sassoon Road, Hong Kong SAR, 00000, China; Division of Life Science, The Hong Kong University of Science and Technology, Clear Water Bay, Kowloon, Hong Kong SAR, 00000, China; Department of Biochemistry and Molecular Genetics, University of Alabama at Birmingham School of Medicine, Birmingham, AL 35294, USA; Division of Life Science, The Hong Kong University of Science and Technology, Clear Water Bay, Kowloon, Hong Kong SAR, 00000, China; Department of Oral and Maxillofacial Surgery, Peking University Shenzhen Hospital, Shenzhen Peking University, Shenzhen, 518036, China; School of Biomedical Sciences, University of Hong Kong, 21 Sassoon Road, Hong Kong SAR, 00000, China; Department of Ocean Science, The Hong Kong University of Science and Technology, Clear Water Bay, Kowloon, Hong Kong SAR, 00000, China; Department of Biochemistry and Molecular Genetics, University of Alabama at Birmingham School of Medicine, Birmingham, AL 35294, USA; Division of Life Science, The Hong Kong University of Science and Technology, Clear Water Bay, Kowloon, Hong Kong SAR, 00000, China; State Key Laboratory of Molecular Neuroscience, The Hong Kong University of Science and Technology, Clear Water Bay, Kowloon, Hong Kong SAR, 00000, China

## Abstract

The six-subunit origin recognition complex (ORC), a DNA replication initiator, defines the localization of the origins of replication in eukaryotes. The Orc6 subunit is the smallest and the least conserved among ORC subunits. It is required for DNA replication and essential for viability in all species. Orc6 in metazoans carries a structural homology with transcription factor TFIIB and can bind DNA on its own. Here, we report a solution structure of the full-length human Orc6 (HsOrc6) alone and in a complex with DNA. We further showed that human Orc6 is composed of three independent domains: N-terminal, middle and C-terminal (HsOrc6-N, HsOrc6-M and HsOrc6-C). We also identified a distinct DNA-binding domain of human Orc6, named as HsOrc6-DBD. The detailed analysis of the structure revealed novel amino acid clusters important for the interaction with DNA. Alterations of these amino acids abolish DNA-binding ability of Orc6 and result in reduced levels of DNA replication. We propose that Orc6 is a DNA-binding subunit of human/metazoan ORC and may play roles in targeting, positioning and assembling the functional ORC at the origins.

## INTRODUCTION

The initiation of cellular DNA replication is tightly controlled event that includes the formation of higher order nucleoprotein complexes at the chromosomal origins of DNA replication (1–4). The eukaryotic initiator, ORC (origin recognition complex), binds to the origins of DNA replication (5) and, working together with the loading factors Cdc6 and Cdt1, recruits the MCM2-7 (minichromosome maintenance) replicative helicase resulting in formation of the pre-replicative complex (pre-RC) at replication origins (6–11). The MCM complex is loaded onto double-stranded DNA as an inactive double-hexamer (12–14) and is activated after origin firing in the S-phase to trigger DNA replication (11,15–17). The exact roles of ORC during the execution of the replication initiation program are long-standing questions in the field.

ORC, a heterohexameric protein complex consisting of Orc1/2/3/4/5/6 subunits, is a critical and conserved component for eukaryotic DNA replication (5,18–21). The Orc1–Orc5 subunits contain AAA+ (ATPases Associated with a variety of cellular Activities) or AAA+-like domains, a subset of which use ATP binding and hydrolysis to support replicative helicase loading, DNA replication and cell viability (22–27). Recently, structural studies showed that the Orc1–Orc5 subunits have a conserved protein structure consisting of one N-terminal AAA+ domain and one C-terminal winged helix domain (WHD) (28–32). A number of reports connect ORC to a variety of human diseases, including Meier–Gorlin syndrome (MGS), diseases resulting from Epstein–Barr virus (EBV) infection, American trypanosomiasis and African trypanosomiasis (33). It has also been shown that Orc1-6 is upregulated in bladder and colorectal cancers (34). However, the exact molecular mechanisms of these disorders still remain unclear in many cases.

The Orc6 protein is the least conserved and perhaps the most enigmatic of all ORC subunits. In budding yeast, Orc6 is essential for viability but is not required for DNA binding *in vitro* (35,36). In *Drosophila*, Orc6 is an integral part of whole ORC and is essential for both DNA binding and replication activity both *in vitro* and *in vivo* (19,25,37). The isolated *Drosophila* Orc6 can bind DNA directly with preference for the poly(dA) sequences (38). In *Xenopus* and humans, Orc6 protein does not seem to be tightly associated with other core ORC subunits (20,39–41). Human Orc6 can bind DNA directly with little or no sequence specificity (41,42). In both, *Drosophila* and human cells, Orc6 has been implicated in coordinating cytokinesis with pre-RC formation and chromosome segregation, a role that it performs independently of the rest of the complex (43–46). Although the full-length human Orc6 structure was not resolved, a conserved C-terminal α helix was found to interact with Orc3 (42,47). Remarkably, the mutations in this motif disrupt the interaction of Orc6 with Orc3 and the rest of the ORC resulting in defects in pre-RC assembly and reduction of MCM2-7 loading in *Drosophila* (47). Specifically, tyrosine 232 to serine mutation in this region of Orc6 is linked to the Meier-Gorlin syndrome, a form of primordial dwarfism in humans (48,49).

Despite the functional diversity of Orc6 in different organisms, it is required for DNA replication and is critical for ORC functions in all studied species (21). Previous structural analysis showed that human Orc6 (HsOrc6) has a homology with transcription factor TFIIB and is able to bind DNA directly (42). However, the structure of full-length HsOrc6 and the detailed mechanism of Orc6/DNA interaction remain elusive. Here, we present the full-length HsOrc6 in *apo* form that contains three independent domains HsOrc6-N (residues 1–94), HsOrc6-M (residues 95–187) and HsOrc6-C (residues 188–252). We also identified HsOrc6 DNA-binding domain, HsOrc6-DBD, spanning residues 95–207. Furthermore, we provide a structural model of HsOrc6–DNA complex based on the experimental NMR data as well as functional studies that demonstrate the importance of HsOrc6-DBD in DNA replication.

## MATERIALS AND METHODS

### Preparation of recombinant proteins and oligonucleotides

Several DNA fragments corresponding to full-length HsOrc6 (residues 1–252), HsOrc6-N (residues 1–94), HsOrc6-M (residues 95–187), HsOrc6-C (residues 188–252), HsOrc6-N+M (residues 1–187), HsOrc6-DBD (HsOrc6 DNA-Binding Domain, residues 95–207) and HsOrc6-N+DBD (residues 1–207) were sub-cloned into the expression vector pET-28a(+) (Novagen), as a fusion with an N-terminal His-tag. All recombinant proteins were transformed into *Escherichia coli* strain BL21 (DE3) for expression. Unlabeled, ^15^N uniformly labeled or ^15^N/^13^C uniformly labeled protein samples were expressed by growing cells in Luria–Bertani (LB) medium or M9 minimal medium (kanamycin 30 μg·ml^-1^) supplemented with ^15^NH_4_Cl and ^13^C-glucose at 37°C overnight after induction at OD_600_ of 0.6–0.8 with 0.5 mM IPTG (Isopropyl β-D-1-thiogalactopyranoside). The cells were harvested by centrifugation and resuspended in buffer containing 50 mM sodium phosphate (pH 7.0) and 300 mM NaCl. The harvested cells were disrupted by sonication, and cell lysate and supernatant were separated by centrifugation at 45 000 × ***g*** for 30 min at 4°C. The supernatant protein was bound to Ni-NTA resin (Qiagen) and subsequently washed by buffer with 10 and 20 mM imidazole, respectively to remove most protein impurities, and then eluted with the same buffer containing 120 mM imidazole. Eluted protein was incubated with 3C protease at room temperature overnight for fusion tag removal. After buffer exchange to remove imidazole, a second Ni-NTA affinity column was applied to separate solubility tags from the tag-free protein products. The tag-free protein was then purified by size-exclusion chromatography (Superdex 75, GE Healthcare Biosciences). The purity of the subsequent eluted protein was detected on a Coomassie-blue-stained sodium dodecyl sulfate (SDS) polyacrylamide gel (PAGE) and shown to be higher than 95%.

DNA oligonucleotides used for NMR were purchased from Integrated DNA Technologies (IDT). The denominations and sequences are 17-bp (5′-GGCCCTTTTTTTTCTAG-3′ and 5′-CTAGAAAAAAAAGGGCC-3′), 12-bp (5′-TTTAAAAAGTAA-3′ and 5′-TTACTTTTTAAA-3′), 11-bp (5′-TTTAAAAAGTA-3′ and 5′-TACTTTTTAAA-3′), 10-bp (5′-TTTAAAAAGT-3′ and 5′-ACTTTTTAAA-3′) and 9-bp (5′-TTTAAAAAG-3′ and 5′-CTTTTTAAA-3′). Double-stranded DNA was prepared by mixing an equal amount of two complementary oligonucleotides in 20 mM sodium phosphate and 30 mM NaCl at pH 6.0, heating to 95°C for 30 min and cooling slowly to room temperature. Double-stranded DNA for NMR experiments was further purified on a Mono-Q 5/50 GL column (Amersham Biosciences) with elution by NaCl concentration gradient from 0.1 to 1 M and changed into the buffer containing 50 mM sodium phosphate (pH 6.5), 100 mM NaCl, 2 mM DTT with 10% ^2^H_2_O or 100% ^2^H_2_O.

### NMR spectroscopy

All NMR spectra were acquired at 298 K on 800-, 750- and 500-MHz Varian NMR spectrometers with cryogenic or room temperature triple resonance gradient probes (750 and 500 MHz). Samples contained 0.1–0.7 mM protein in 50 mM sodium phosphate (pH 6.5), 100 mM NaCl, 2 mM DTT with 10% ^2^H_2_O added for the lock or 100% ^2^H_2_O. For protein–DNA complex samples, 0.5 mM protein and 2-fold of DNA were incubated in the same buffer of free protein. All spectra were processed using NMRPipe (50,51) and analyzed using SPARKY 3 (Goddard and Kneller, University of California, San Francisco, CA, USA). Protein backbone assignments for ^15^N, ^1^HN, ^13^Cα, ^13^Cβ and ^13^CO chemical shifts were obtained from HNCACB, CBCA(CO)NH, HNCA and HNCO experiments (52). Amino acid side chain resonance assignments were obtained from standard ^1^H-^15^N TOCSY-HSQC, CC(CO)NH, HBHA(CO)NH (53), HCCH-TOCSY, ^15^N- and ^13^C-edited NOESY-HSQC experiments (52). Aromatic resonances were assigned using 2D ^1^H–^13^C HSQC, HBCBCGCDHD, HBCBCGCDCEHE (54) and NOESY spectra. The protein–DNA complex samples were prepared at a 1:2 ratio between ^15^N, ^13^C-labeled protein and 17-,12-bp or 10-bp DNAs. NOE-derived distance restraints were obtained from ^15^N- or ^13^C-edited 3D NOESY spectra each with a mixing time of 120 ms for free protein. For the intermolecular contact, ^13^C-edited, ^13^C/^15^N-filtered 3D NOESY spectra were recorded (mixing time: 150 ms) (55). To study the dynamical properties of the protein alone or bound with DNA, NMR data were recorded at 298 K for 0.15 mM of Orc6-DBD (residues 95–207) protein with and without a 17-bp DNA (ratio 1:2 of protein to DNA) on 500 and 800 MHz NMR spectrometer at 298 K. Steady-state heteronuclear [^1^H]–^15^N-NOE experiments were performed as described (56) and recorded with and without 3 s of ^1^H saturation. Amide ^15^N transverse relaxation rates (*R*_2_) were measured using CPMG delays containing two redundant delays: 10, 30, 50/50, 70, 90, 110/110, 130 and 150 ms. ^15^N longitudinal relaxation rates (*R*_1_) were measured using inversion recovery delays with two redundant delays: 100, 300, 500/500, 700, 900, 1100/1100, 1300 and 1500 ms. Duplicate time points were used for error estimation. The values of *R*_1_ and *R*_2_ were obtained by fitting the extracted peak intensities to a mono-exponential decay curve. Peak intensities were extracted using the relaxation module in SPARKY 3 (Goddard and Kneller, University of California, San Francisco, CA, USA) and data fitting was performed by Curvefit (A. G. Palmer, Columbia University) using the script ‘sparky2rate’ (http://ursula.chem.yale.edu/∼lorialab/sparky2rate). The correlation time (τ_c_) of the protein molecule was then estimated using the ratio of averaged *R*_2_/*R*_1_ values using the residues within helical segments (57).

### NOE analysis and structure calculations

Nuclear Overhauser effect (NOE) assignment and structure calculations were performed using the program CYANA2.1 (58). The final set of NOE distance restraints derived from CYANA together with H-bond restraints as well as the φ and ψ backbone dihedral angle restraints derived from TALOS+ (59) based on the chemical shifts were used for molecular dynamics simulated annealing and water refinement by using the program of RECOORD (60). The quality of the structures was assessed using PROCHECK-NMR (61) and analyzed by MOLMOL (62). The solvent accessibility was calculated by NACCESS (63). All of the figures representing the structures were generated by Pymol (http://www.pymol.org). The statistics of the structure refinement and the quality of the final structures are summarized in Supplementary Table S1 for the full-length HsOrc6 in apo form. The atomic coordinates have been deposited at the Protein Data Bank with accession code 6KVG.

### HADDOCK docking

The information drive docking program HADDOCK 2.2 (64) was used to generate the HsOrc6–DNA complex model. The starting structure for docking was a B'-form model of 10-bp DNA (5′-TTTAAAAAGT-3′ and 5′-ACTTTTTAAA-3′) constructed using the 3DNA (65) based on the known structure of poly(dA).poly(dT) (66) and the lowest energy structure of the full-length HsOrc6. The residues with chemical shift perturbations of amide resonances >0.038 parts per million (ppm) and with high solvent accessibility (>50%) were selected as active residues and the neighbors of these active residues were selected as passive residues. For DNA, THY1 to ADE8 and THY13 to ADE20, which were all highly affected by titrating proteins, were selected as active bases. No passive residues were selected for DNA. Side-chains of all selected HsOrc6 residues were allowed to move freely during the semi-flexible refinement process, except side chains of HsOrc6-C domain (residues 188–252) were set to fully flexible in this refinement process. To maintain the DNA structure, the whole DNA molecule was set as rigid and the NOE distances derived from 2D ^1^H-^1^H NOESY spectra of free and protein-bound DNA were added during the final water refinement. As the residues Arg198 to Lys201 of HsOrc6-C domain are completely flexible and their conformation is independent of other parts of HsOrc6, a flexible multidomain docking protocol, a ‘divide-and-conquer’ approach, was followed to model the HsOrc6–DNA complex in HADDOCK2.2 (67). A total of 1000 structures were generated during rigid body energy minimization, and 200 structures with the lowest energy were selected in the semi-flexible refinement process. These 200 structures were finally refined using an explicit solvent of water and followed by clustering using backbone rmsd for both protein and DNA structures by a cutoff of 8.5 Å with a minimum of 10 structures in each cluster, which yielded five clusters. The lowest energy structures from the first cluster were selected and used for representation of the HsOrc6–DNA complex.

### NMR titrations

^1^H, ^15^N HSQC spectra of protein titrated with DNA were carried out to map the DNA-binding region of the complex. About 0.1 mM ^15^N-labeled protein was titrated with increasing amounts of DNA with various ratios of DNA to protein ranging from 0.1 to 0.6 mM. All measurements were acquired on a 750 MHz Varian NMR spectrometer at 298 K using the NMR buffer described above. DNA oligonucleotides used for NMR titration were purchased and prepared as described above. The weighted chemical shift perturbations for backbone ^15^N and ^1^HN resonances were calculated by the equation Δδ = [[(ΔδHN)^2^+(ΔδN/5)^2^]/2]^0.5^, where ΔδHN and ΔδN are the differences in chemical shifts of amide protons and nitrogen between the initial and final data points of the titration, respectively.

For 2D ^13^C/^15^N-filtered ^1^H-^1^H NOESY experiments of the 10-bp DNA, the NMR sample contained 0.5 mM DNA in 50 mM potassium phosphate, 100 mM NaCl (pH6.5) in 100% D_2_O, with or without 1.0 mM protein. The 2D ^13^C/^15^N-filtered ^1^H-^1^H NOESY spectra of free DNA and in complex with protein were collected on a Varian 800 MHz spectrometer with a triple-resonance cryoprobe at 298 K (55). The DNA/protein complex sample was prepared by dissolving lyophilized protein (1.0 mM final concentration) into the DNA sample. The fingerprint region of intra-residue H1′-H6/ H8 NOE peaks was analyzed, and the peaks were assigned as previously described (68).

### Measurement of dissociation binding constants by NMR

The dissociation constant (*K*_D_) for DNA binding to Orc6 was calculated by plotting chemical shift changes as a function of the DNA-to-protein ratio and then fitting the values to a function using the curve-fitting software, xcrvfit (www.bionmr.ualberta.ca/bds/software/xcrvfit). The function relating the predicted change in chemical shift to total protein (*P*) and total DNA concentrations (*D*) is as follows:

$$\begin{equation*}\Delta \delta \ = \Delta {\delta _{max}}\ \left[ {\frac{{P + D + {K_D} - \sqrt {{{\left( {P + D + {K_D}} \right)}^2} - 4PD} }}{{2P}}} \right]\end{equation*}$$

where $\Delta {\delta _{max}}$ is the change in chemical shift expected at 100% saturation and *K*_D_ is the dissociation constant for the 1:1 protein–DNA complex. A χ^2^ function measuring the sum of differences between observed and predicted $\Delta \delta$ values was minimized, using *K*_D_ and $\Delta {\delta _{max}}$ as fitting parameters (69).

### Isothermal titration calorimetry (ITC)

Thermodynamic attributes of the interaction profiles between HsOrc6 and DNA sequences are analyzed by ITC using iTC200 Microcalorimeter at 25°C. The HsOrc6 and HsOrc6-4A proteins are diluted to 20 μM in 50 mM sodium phosphate containing 100 mM NaCl (pH 6.5). Syringe is filled with 500 μM DNA dissolved into the same buffer. The heat of reaction per injection (μcal/s) is determined by integration of the peak areas using in-built Origin 7.0 software. Data points are further simulated with ‘one-site’ binding modes.

### DNA-binding assay

All mutants were created by site-directed mutagenesis following standard protocol (Stratagene), cloned into pET-15b plasmid and expressed in BL21 cells. His-tagged HsOrc6 wild-type or mutant proteins were purified using His-Pur Cobalt Resin (Thermo scientific) and cation exchange Hi-Trap SP HP column (GE Healthcare 17-1151-01).

For the electrophoretic mobility shift assay (EMSA), the binding reactions were carried out in 10 μl of 25 mM Tris, pH 8.0/60 mM KCl/5 mM MgCl_2_/0.4 mM EDTA/0.4 mM EGTA/0.1% NP-40 (Octylphenoxy poly(ethyleneoxy)ethanol)/10% glycerol/0.12 mg/ml BSA. Each reaction contained ∼150 ng of purified protein, 50 or 100 ng of Poly(dGdC) competitor and 1 ng of ^32^P end-labeled Lamin B2 DNA. Reactions were set up on ice and incubated at room temperature for 30 min. Each reaction was loaded on a 4.5% polyacrylamide gel 50:1 (acrylamide:bis-acrylamid). Electrophoresis was performed in 1× TAE buffer pH 9.3. Gel was dried on Whatman paper and exposed to X-ray film.

### DNA replication assay

DNA replication assay in *Xenopus* extracts was performed based on the procedures previously described (42,70–72). In brief, the endogenous *Xenopus* Orc6 protein was depleted from the extracts using antibodies raised against human and *Drosophila* Orc6 proteins as in (42). Next, Orc6-depleted extracts were supplemented with recombinant proteins to verify the activity of the human wild-type and mutant Orc6 in DNA replication. For replication assays, extracts (50 μl) were supplemented with demembranated *Xenopus* sperm nuclei (71) to give a final DNA concentration of 2–5 ng/ml, in the presence of [α^32^P]dCTP. After 30 min of incubation at 23°C, DNA was extracted and ethanol precipitated, resuspended and submitted to the electrophoresis in a 0.8% agarose gel. The gel was dried and autoradiographed.

### Immunostaining of salivary glands polytene chromosomes

GFP fused, wild-type HsOrc6 and HsOrc6 mutants were cloned under the UAS promoter in the pUAST vector and injected into fly embryos. Homozygous fly stocks were set up. To induce GFP-HsOrc6 expression in salivary glands, female flies from established stock were crossed to males bearing GAL4 driven by *sgs3* promoter (Bloomington *Drosophila* Stock Center cat- number 6870). *Sgs3* promoter induces GAL4 in salivary glands of third instar larvae. Salivary glands of these larvae were dissected in PBS supplemented with 0.5% NP-40, fixed for 1 min in 2% formaldehyde and squashed in 45% acetic acid. Slides with chromosome squashes were frozen in liquid nitrogen and desiccated in 96% ethanol two times. For antibody application slides were washed in PBS, incubated with anti GFP rabbit polyclonal primary antibodies (Abcam ab290) for 2 h, washed in PBS two times for 10 min, incubated with secondary antibodies Alexa Fluor 488, stained with DAPI and analyzed under Olympus BX61 fluorescent microscope.

To verify the expression of GFP-HsOrc6 proteins, four pairs of salivary glands were isolated from corresponding fly stocks and homogenized in a loading buffer. The proteins were separated by SDS-gel electrophoresis and analyzed by Western blotting using anti-GFP monoclonal antibodies (B2, Santa Cruz Biotech). The same blot was incubated with Pnut polyclonal antibodies to justify loading level. Two independent fly stocks were analyzed for each mutant.

## RESULTS

### The three domains of HsOrc6 are independent structural modules

**Figure 1.**
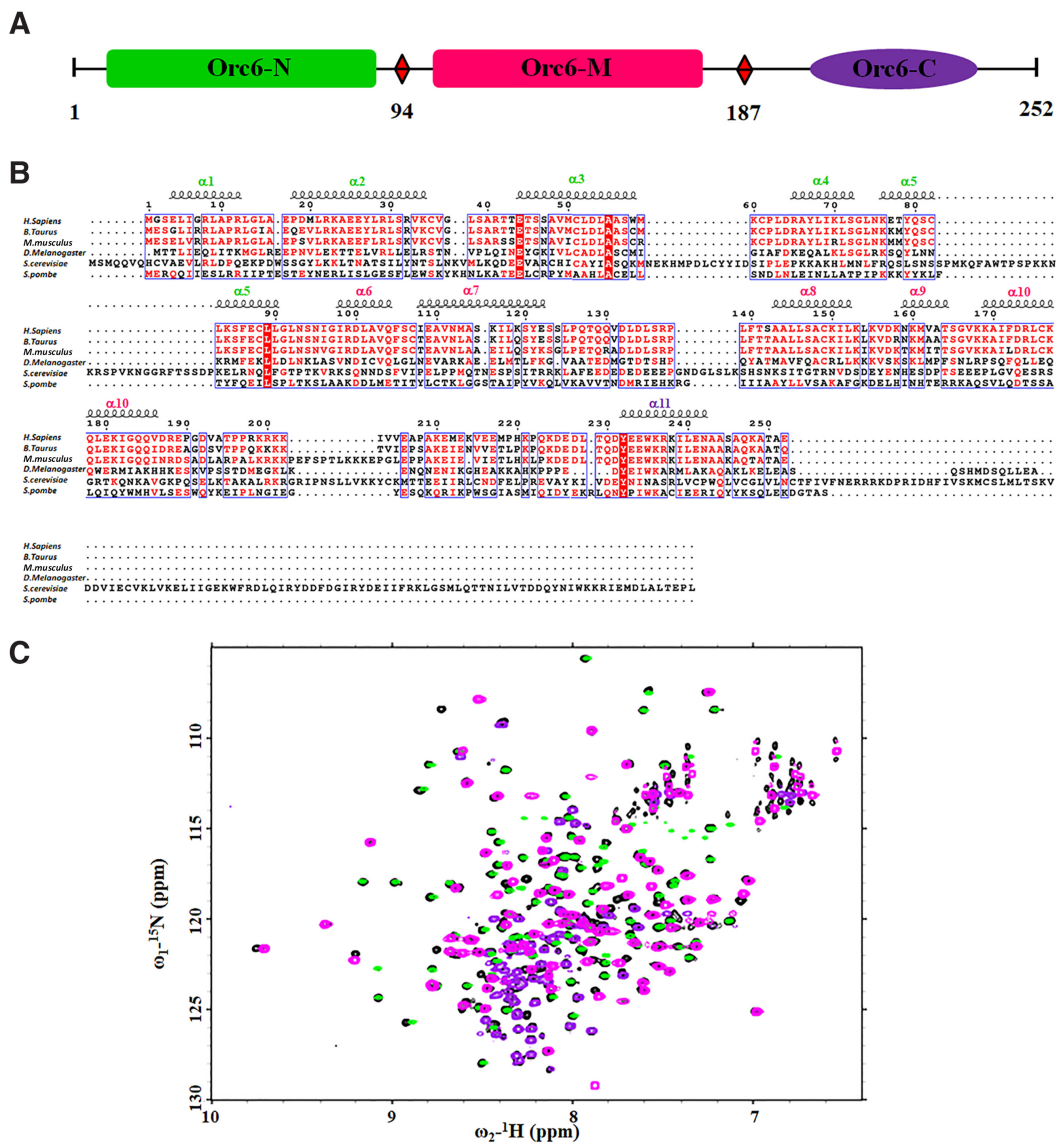
(**A**) Schematic representation of the full-length human Orc6. (**B**) Sequence alignment of the Orc6 sequences from different species: Q9Y5N6, *Homo sapiens*; Q2HJF3, *Bos taurus*; Q9WUJ8 *M. musculus*; Q9Y1B2, *Drosophila melanogaster*; P38826, *Saccharomyces cerevisiae*; O74796, *Schizosaccharomyces pombe*. The dashes indicate the positions of gaps in eukaryotic sequences. The sequence alignment was produced with ClustalW2 (73) and plotted with ESPript 2.2 (74). The indicated secondary structure corresponds to the solution structure of full-length human Orc6 reported here. Residues are red-scaled based on percentage identity. (**C**) The superposition of ^1^H,^15^N HSQC NMR spectra of individual Orc6-N(green), Orc6-M (magenta), Orc6-C (purple) and full-length Orc6 (black) are shown.

HsOrc6 encompasses 252 amino acids and is predicted to consist of two smaller globular domains connected with a short linker region and a helical extension that engages a short α-helix formed at the C-terminus that has been characterized by previous studies (Figure 1A) (42). The sequences from different species are compared and aligned by ClustalW2 (73) and by ESPript (74) (Figure 1B and Supplementary Figure S1). Three HsOrc6 constructs, HsOrc6-N (residues 1–94), HsOrc6-M (residues 95–187) and HsOrc6-C (residues 188–252) were designed according to previously described studies (42). A superposition of ^1^H, ^15^N-HSQC spectra of the three individual HsOrc6 domains and full-length HsOrc6 is shown in Figure 1C. The chemical shifts of the NMR signals in the individual HsOrc6 domains are very similar compared with the full-length HsOrc6 construct with the exception of the chemical shifts of residues located at the boundaries of the isolated domains (Supplementary Figure S2). These data indicate that the three individual domains in HsOrc6 are largely independent structural modules. Furthermore, there were no chemical shift perturbations observed when performing NMR titration experiments indicating that there is no evidence of strong domain–domain interaction (Supplementary Figure S3).

### Overall structure of full-length HsOrc6 in apo form

**Figure 2.**
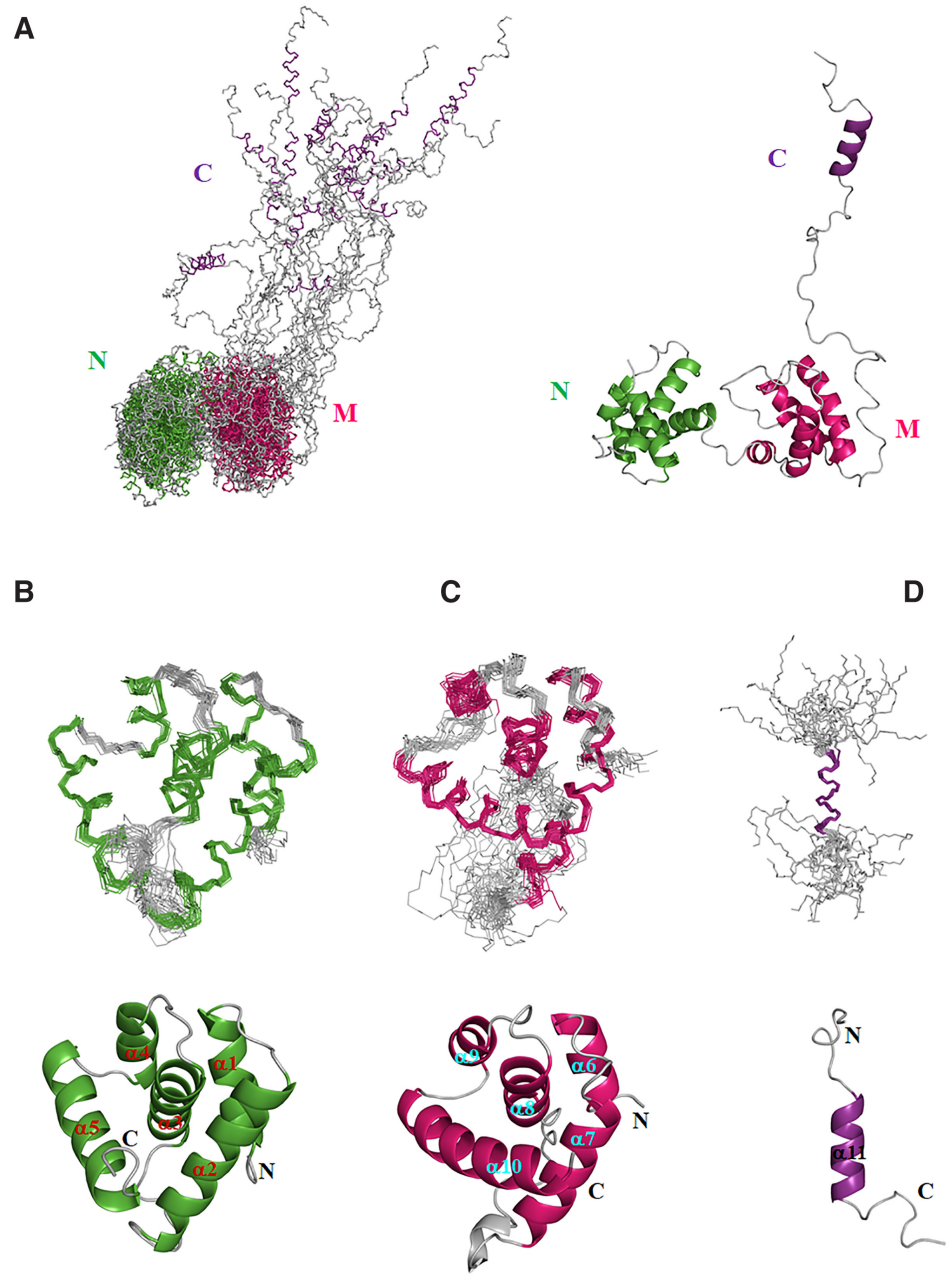
(**A**)The ensemble of the lowest 20 energy structures of full-length Orc6 generated by superposition of backbone atoms of residues 1–252 (left) and the lowest structure of the ensemble shown in cartoon (right). Individual (**B**) Orc6-N (residues 1–94, green), (**C**) Orc6-M (residues 95–187, magenta) and (**D**) Orc6-C (residues 222–252, purple) domains are shown in superimposed ensemble and in a form of cartoon of the lowest structure. The secondary structure of each domain is colored as green in Orc6-N, magenta in Orc6-M and purple in Orc6-C. The loop in each domain is shown in gray. The region containing residues from 188 to 221 is flexible and is not shown in (D).

To understand the role of HsOrc6 in DNA replication, we solved the solution structure of the full-length HsOrc6 using NMR. NMR resonance assignment of the full-length protein is described briefly in ‘Materials and Methods’ section. All backbone resonances were clearly identified except for those of Leu135, Ser136 and Arg137 (Supplementary Figure S4) and approximately 90% of the side chain resonances were unambiguously assigned. The ensemble of the 20 lowest energy structures of full-length HsOrc6 after water refinement is shown in Figure 2A. Structural statistics are summarized in Supplementary Table S1. There were no inter-domain NOEs observed, confirming our observation that the three domains do not interact. Therefore, the three domains were analyzed individually and presented in Figure 2B–D. The structures of HsOrc6-N, HsOrc6-M and HsOrc6-C are well defined by NMR data except for the C-terminal residues 188–230 and 243–252, which are not structured. These C-terminal residues show no long-range NOEs to the rest of the protein and have low backbone [^1^H]-^15^N heteronuclear NOEs, indicative of the high mobility in solution (Figure 2A and Supplementary Figure S5).

The structures of the HsOrc6-N and HsOrc6-M domains reveal a compact architecture that is built on a central helix (α3 in HsOrc6-N and α8 in HsOrc6-M) connecting two helix-turn-helix motifs at the N- and C- termini of the individual domain (α1-turn-α2 and α4-turn-α5 of Orc6-N; α6-turn-α7 and α9-turn-α10 of HsOrc6-M) (Figure 2B and C). In HsOrc6-N domain, the α1-turn-α2 motif is packed against one side of α3 and the α4-turn-α5 is located on the opposite side of α3. A similar packing mode is observed for the α6-turn-α7 and α9-turn-α10 motifs in HsOrc6-M domain. Many hydrophobic contacts were observed between the central helix and helix-turn-helix motifs such as Ile6 and Leu9 of α1, Tyr27 and Leu30 of α2, Val49 and Leu54 of α3, Tyr67 and Ile69 of α4 and Tyr79 and Phe86 of α5 in HsOrc6-N domain. Similar hydrophobic contacts in HsOrc6-M domain were also observed such as Ile98 and Phe105 of α6, I117 and Leu118 of α7, Alal44 and Ile151 of α8, Val162 of α9 and Phe172 and Leu179 of α10 (Supplementary Figure S6A and S6B). The fold of the HsOrc6-N and HsOrc6-M domains is almost the same except for the difference in angle (∼20°), between α3 in Orc6-N and α8 in Orc6-M (Supplementary Figure S6C). The structure of HsOrc6-M domain almost matches the previously reported crystal structure except the linker between α7- and α8-containing residues Gln127-Leu133 that forms a helix in the X-ray structure (Supplementary Figure S6D) (42). This helix was not well established in the solution structure possibly due to the high flexibility of the linker connecting α7 and α8. However, the HsOrc6-C domain adopts an amphipathic α-helical conformation (Figure 2D) containing residues 231–242 that is consistent with the observation in the crystal structure of the *Drosophila* ORC (28).

### Binding of HsOrc6 to a poly-AT rich DNA sequence

**Figure 3.**
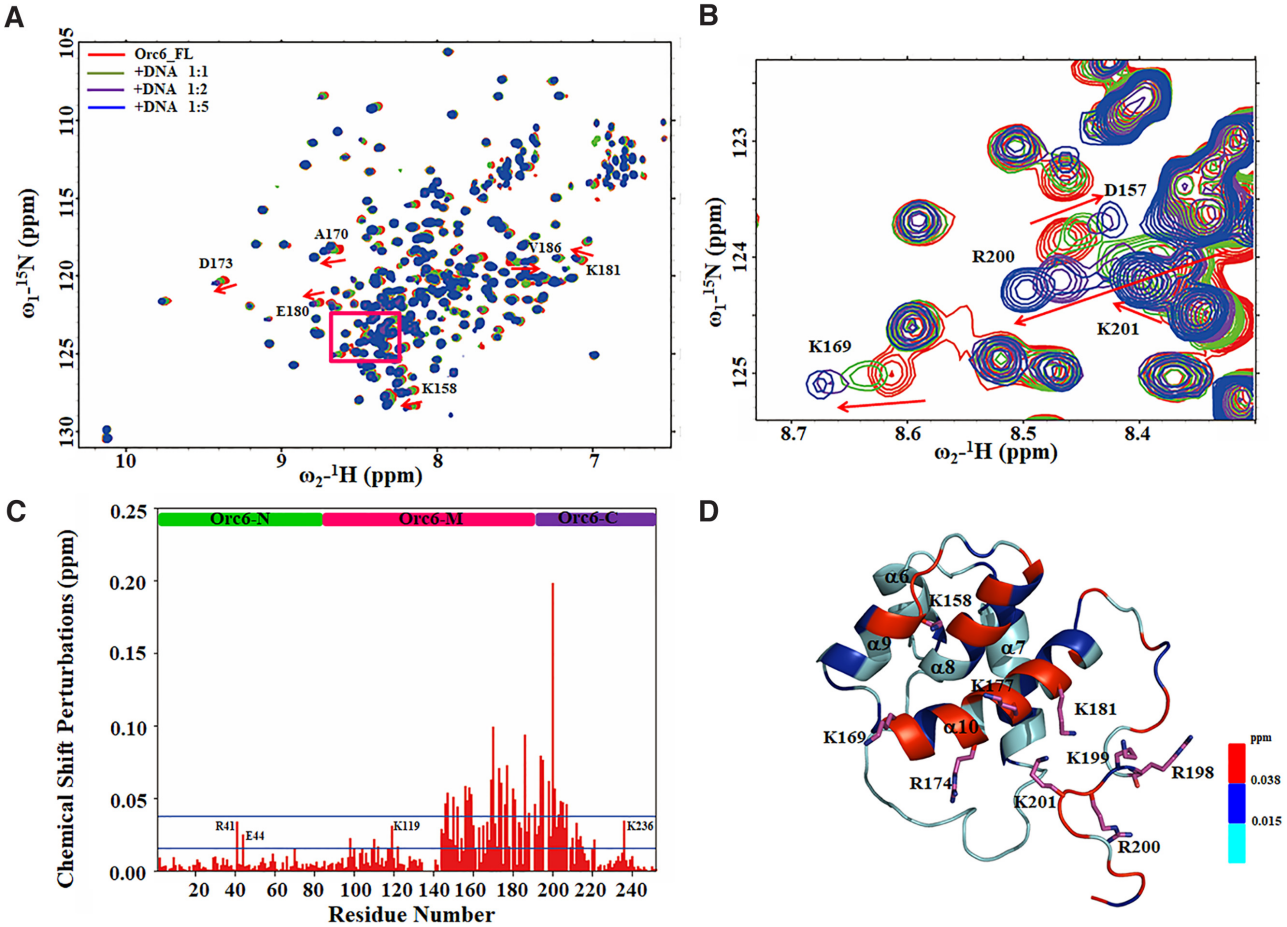
NMR study of the interaction between Orc6 and DNA. (**A**) The overlaid ^1^H,^15^N-HSQC spectra of full-length Orc6 in free form (red) titrated with 17b DNA at a molar ratio of 1:1 (green), 1:2 (purple) and 1:5 (blue). Residues that undergo significant changes in chemical shifts upon formation of the complex with DNA are indicated with arrows and labeled with peak assignments. (**B**) A zoomed part of overlaid ^1^H,^15^N-HSQC spectra shown in (A) with red square. (**C**) Weighted chemical shift perturbations for backbone ^15^N and ^1^HN resonances as calculated by the equation Δδ = [(ΔδHN)^2^+(ΔδN/5)^2^]^0.5^. The mean Δδ value (0.015 ppm) and the mean Δδ value plus 1 SD (0.038 ppm) of the chemical shift perturbations are plotted as solid lines. (**D**) Chemical shift perturbations in the presence of 17bp DNA are colored onto the structure of HsOrc6-DBD (resides 95–207) in ribbon representation. Residues with chemical shift perturbations ranging from 0.015 to 0.038 ppm are colored in blue, whereas residues with chemical shift perturbations larger than 0.038 ppm are shown in red. The residues, Lys158, Lys169, Arg174, Lys177, Lys181, Lys198, Arg199, Lys200 and Arg202, are shown in the stick model.

A previous study showed that HsOrc6, the smallest ORC subunit, is a DNA-binding protein that is necessary for the DNA binding and DNA replication functions of ORC. HsOrc6 binds DNA fragments containing the origins of DNA replication and prefers poly(dAT) sequences (41,42). To characterize the DNA-binding properties of the HsOrc6, amide chemical shifts were monitored upon titration of a 17bp DNA oligonucleotide to the full-length ^15^N-labeled HsOrc6 (Figure 3A and B). The weighted chemical shift perturbations of backbone amide resonances (Figure 3C) were calculated and mapped onto the HsOrc6 structure (Figure 3D). The backbone amide resonances with significant chemical shift perturbations on DNA binding (Δδ > Δδ_average_ + SD ∼ 0.038 ppm) are mostly located at the α8, α9 and α10 helices of HsOrc6-M domain (Figure 3D). Notably, the residues R198, K199, R200 and K201 of Orc6-C domain show the most marked chemical shift perturbations, suggesting that this C-terminal region may be involved in direct interaction with DNA or undergo major conformational changes upon DNA binding. We used the titration curves for HsOrc6 residues with significant chemical shift perturbations to determine the *K*_D_ values of DNA binding (Supplementary Figure S7). The *K*_D_ values we measured ranged from 36.4 ± 1.3 to 54.3 ± 5.2 μM.

Intriguingly, it seems that HsOrc6-N is not involved in binding with DNA. To confirm the DNA-binding surface in Figure 3D, the individual Orc6 domains, HsOrc6-N, HsOrc6-M and HsOrc6-C were titrated with 17bp DNA. As shown in Supplementary Figure S8A, no chemical shift differences were observed for HsOrc6-N domain indicating that Orc6-N domain alone does not bind to DNA. The HsOrc6-M domain exhibits similar chemical shift changes upon addition of DNA when compared to the full-length HsOrc6 with DNA (Supplementary Figure S8B). However, the HsOrc6-C domain shows weak interactions with DNA (Supplementary Figure S8C). The HsOrc6 fragment containing residues 95-207, termed as HsOrc6 DNA-binding domain (HsOrc6-DBD) was used to do NMR titration with DNA. Interestingly, similar chemical shift perturbations are observed for HsOrc6-DBD upon saturated binding to DNA when compared to full-length HsOrc6 titrated with DNA with the *K*_D_ ranging from 9.9 ± 2.9 to 57.7 ± 1.1 μM (Supplementary Figure S9). Based on the NMR spectral changes, all these data indicate that the binding regions of HsOrc6 with DNA comprise the recognition helices α8, α9 and α10 and the C-terminal amino acids Arg198-Arg201. The larger chemical shift changes of the C-terminal residues in the full-length Orc6 compared to the individual HsOrc6-C domain may be due to the cooperative binding effects. Furthermore, the interaction of HsOrc6 and HsOrc6-N with DNA was measured by ITC method resulting in *K*_D_ consistent with the observation by NMR (Supplementary Figure S10).

It is worth noting that several positively charged residues Arg41, Glu44, K119 and K236 of full-length HsOrc6 also show large chemical shift changes when titrated with DNA suggesting the presence of non-specific charge interaction with DNA (Figure 3C).

### The shortest DNA fragment required for the interaction with HsOrc6

Although the binding affinity of the full-length HsOrc6 with 17 bp DNA is around 10^−5^ M, we failed to detect intermolecular NOEs by ^13^C-edited, ^13^C/^15^N-filtered 3D NOESY experiments on the full-length Orc6 complex with 17bp DNA (55). We speculated that the HsOrc6-M and HsOrc6-DBD (residues 95–207) domains of HsOrc6 could provide information on the intermolecular NOEs. However, the intermolecular NOEs were still missing. The failure to detect intermolecular NOEs is possibly due to the non-specific binding of DNA by Orc6 (41). As *Drosophila* Orc6 can bind DNA with the preference for the poly(dA) sequences (37,38), we measured the binding affinity of HsOrc6-DBD to AT-rich DNAs of different length derived from 17bp DNA. To resolve the severe overlap in the H1’-H6/H8 region of 2D NOESY spectrum, five T bases were changed to A bases. As shown in Supplementary Figures S11 and S12, the shortest length for AT-rich DNA that can be bound by HsOrc6-DBD is 10 bp. Therefore, we hypothesize that DNA binds with HsOrc6 with a 1:1 ratio based on the size of the HsOrc6-DBD. The hypothesis for the stoichiometry of HsOrc6-DBD/DNA complex was verified by the analysis of ^15^N relaxation data for HsOrc6-DBD in the DNA free and bound state (Supplementary Figure S13). Average rotational correlation times (τ_c_) were estimated for HsOrc6-DBD and HsOrc6-DBD/17bp-DNA from the ratio of ^15^N *R*_2_/*R*_1_ relaxation rates assuming isotropic rotational diffusion (57). This analysis shows τ_c_ values of ∼8.9 ns for HsOrc6-DBD alone and ∼13.7 ns for HsOrc6-DBD in the presence of DNA. A rotational correlation time of ∼14 ns would be expected for a 23.2 kDa HsOrc6-DBD/DNA complex (a ratio of 1:1), whereas ∼8 ns expected for HsOrc6-DBD alone (12.8 kDa). Therefore, a model of HsOrc6/DNA complex in ratio 1:1 can be built based on the chemical shift perturbations.

**Figure 4.**
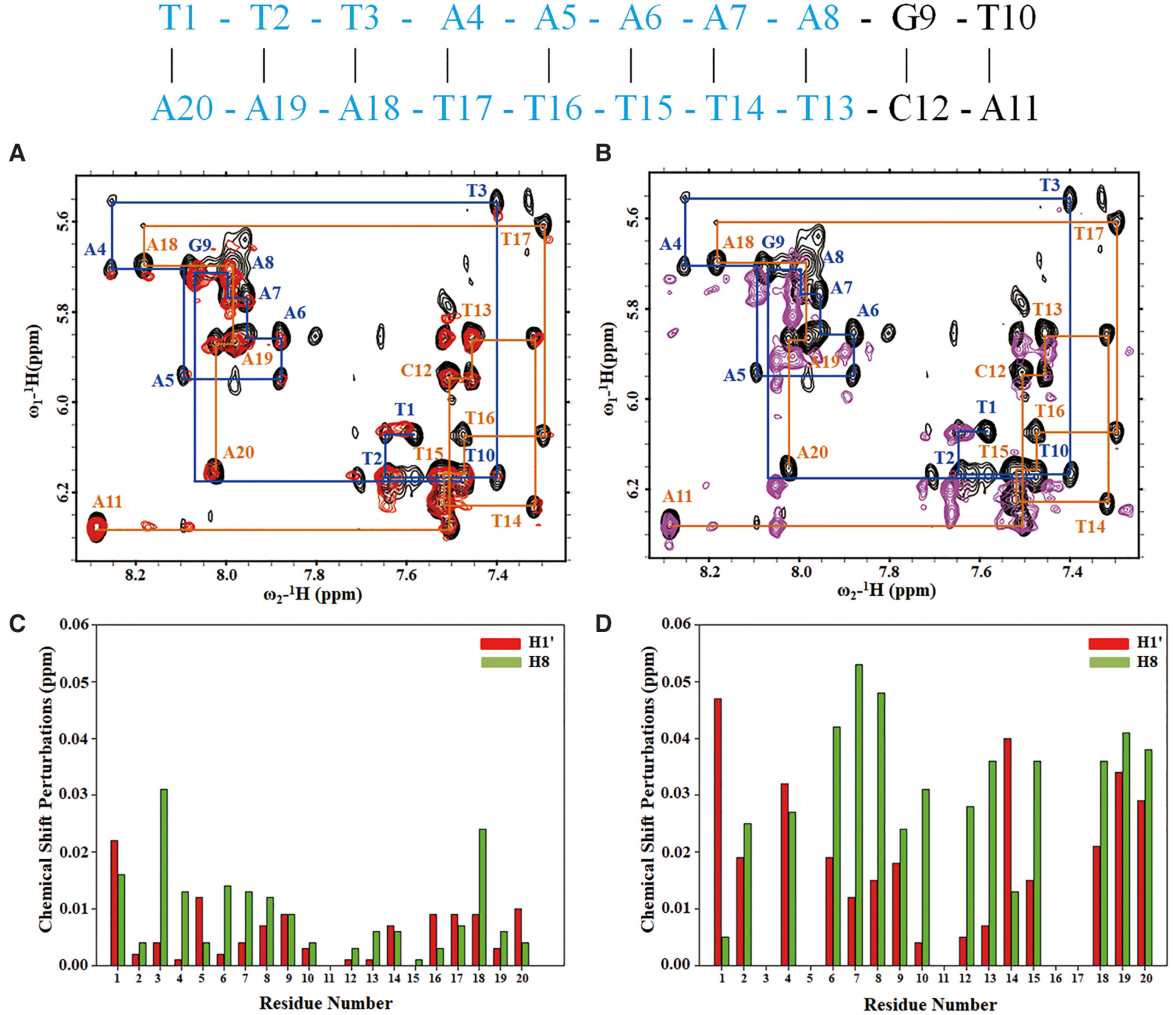
(**A** and **B**) Overlay of the fingerprint region showing intraresidue H1′–H6/H8 NOE peaks of 2D ^13^C/^15^N-filtered ^1^H-^1^H NOESY spectra of free (black) and Orc6-M bound with DNA (red) and HsOrc6-DBD (residues 95–207) bound with DNA (purple) at concentration ratio 1:2 (DNA, 0.5 mM: HsOrc6-DBD,1.0 mM). Intraresidue H1′–H6/H8 NOE peaks of free DNA are labeled by base type and number. The sequence the 10-mer DNA is shown above, with residues affected by Orc6 binding indicated in blue. (**C** and **D**) ^1^H chemical shift difference (Δδ) for H1′ (red) and H6/H8 (green) chemical shifts between free and Orc6-bound DNA corresponding to (A) and (B) are plotted against residue number.

To map the binding interface on DNA, we analyzed chemical shift changes of the H1′ and H6/H8imino protons in the 10-bp AT-rich DNA duplex upon addition of the HsOrc6-M and Orc6-DBD, respectively (Figure 4). Comparison of 2D ^1^H-^1^H NOESY spectra of the DNA, free or in complex with Orc6, reveals that residues with significant intra-residue H1′-H6/H8 NOE peak shift (H1′ or H6/H8 Δδ > 0.025 ppm) are T1-T3, A4-A8, T13-T17 and A18–A20 (Figure 4C and D). This provides further evidence for the preference of Orc6 in the binding to AT-rich regions. The larger chemical shift changes after the addition of HsOrc6-DBD compared with the addition of HsOrc6-M demonstrated that the new DNA-binding sites are located within the HsOrc6-C domain. Backbone [^1^H]-^15^N heteronuclear NOE measurements (Supplementary Figure S13D) confirm that the region containing residues 190–207 is highly flexible in the free protein but becomes more ordered in the presence of DNA (NOE values increased from ∼-0.5 to ∼0.0).

### *In vitro* and *in vivo* studies of the interaction between HsOrc6 and DNA

**Figure 5.**
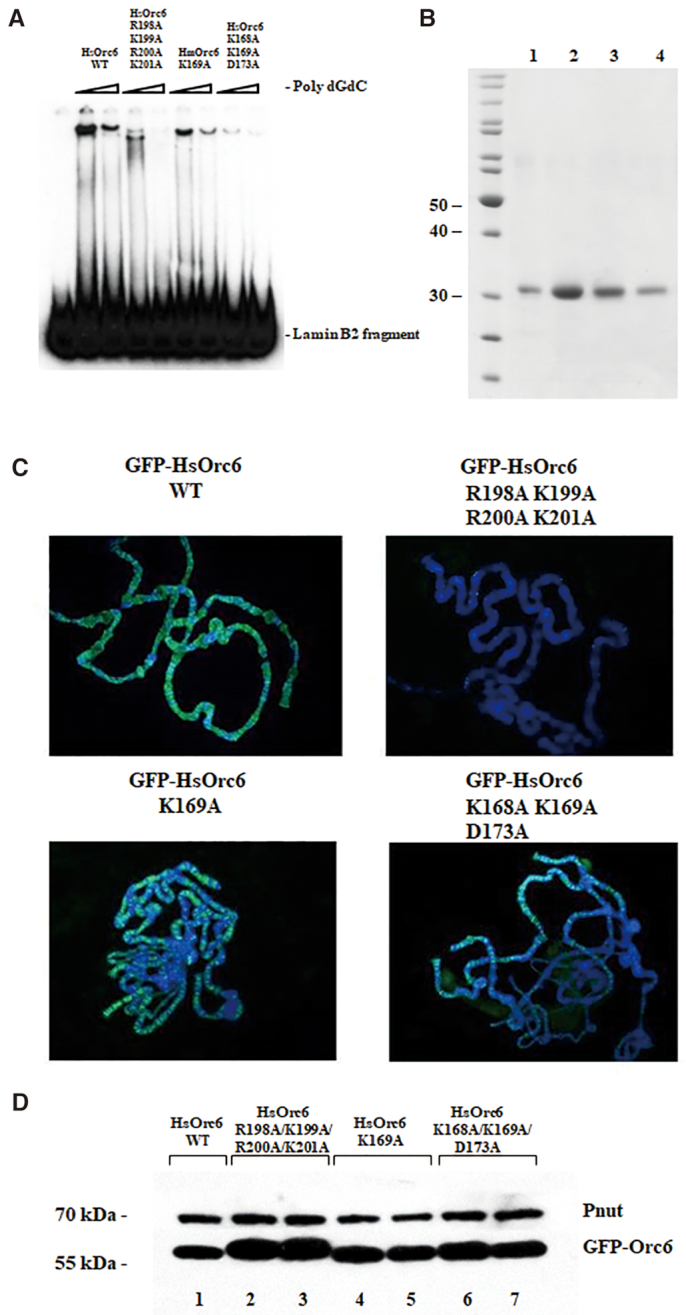
DNA-binding ability of HsOrc6. (**A**) DNA binding of human Orc6 wild-type HsOrc6WT and mutants HsOrc6R198A/K199A/R200A/K201A, HsOrc6K169A, HsOrc6K168A/K169A/ D173A to radiolabeled origin fragment Lamin B2 in the presence of poly dGdC competitor DNA was monitored by EMSA. The amount of competitor was 50 and 100 ng. (**B**) Silver stained gel of purified wild-type human Orc6 (1) and mutants HsOrc6R198A/K199A/R200A/K201A (2), HsOrc6K169A (3), HsOrc6K168A/K169A/D173A (4). (**C**) Immunostaining of GFP- fused wild-type and mutant Orc6 proteins expressed in salivary glands of *Drosophila* third instar larvae. Orc6 was detected with anti-GFP antibodies. (**D**) The expression level of the GFP-tagged Orc6 proteins in salivary glands of fly strains used in (C). Salivary glands of *Drosophila* larvae expressing GFP-tagged Orc6 proteins were isolated and homogenized. The proteins in the extracts were separated by SDS-gel electrophoresis and analyzed by Western blotting using anti-GFP polyclonal antibodies. HsOrc6WT (lane 1), HsOrc6-R198A/K199A/R200A/K201A (lanes 2 and 3), HsOrc6-K169A (lanes 4 and 5), HsOrc6-K168A/K169A/D173A (lanes 6 and 7) are shown. Pnut protein was used as a loading control.

Based on our data presented here, two amino acid clusters were selected for mutagenesis (198–201 and 168–173). Three HsOrc6 mutants were created: HsOrc6-K169A, HsOrc6-K168A/K169A/D173A and HsOrc6-R198A/K199A/R200A/K201A. Interestingly, these amino acid clusters encode a perfect nuclear localization signal that is often associated with the protein sequences responsible for DNA recognition (75,76) (Supplementary Table S2). Mutant HsOrc6 proteins were expressed in *E. coli*, purified (see ‘Material and Methods’) and tested for DNA binding using electrophoretic mobility shift assay (EMSA). We found that the wild-type HsOrc6 formed a distinct complex with Lamin B2 DNA fragment; however, the binding abilities of HsOrc6-K168A/K169A/D173A and HsOrc6-R198A/K199A/R200A/K201A mutants were compromised as shown in Figure 5A. Mutant proteins expressed and purified undistinguishable from the wild-type HsOrc6 protein (Figure 5B). NMR analysis of the HsOrc6 protein containing triple alanine mutations did not reveal significant differences as compared to the wild-type protein (Supplementary Figure S14). Taken together, these data suggest that the inability of the mutants to bind DNA was not due to structural changes caused by the mutations.

HsOrc6 associates with chromatin in human cell lines (44,77). Moreover, HsOrc6 partially restores DNA replication when expressed in *Drosophila* mutant cells carrying the deletion of Orc6 gene (38). In order to visualize the binding of HsOrc6 mutant proteins to the chromosomes *in vivo*, we expressed them in *Drosophila* salivary glands. Nuclei of *Drosophila* salivary glands contain polytene chromosomes that can easily be visualized with microscopy because of their giant size and well-determined structure. GAL4-UAS system is a commonly used genetic tool that allows to tissue-specifically overexpress gene of interest in *Drosophila* (78). Using this method, we overexpressed HsOrc6 proteins specifically in salivary glands. GFP-fused wild-type HsOrc6 protein was tightly associated with polytene chromosomes (Figure 5C) as we have shown previously (37,42). In contrast, HsOrc6 mutant HsOrc6-R198A/K199A/R200A/K201A failed to associate with chromosomes (Figure 5C) in agreement with *in vitro* DNA-binding experiments shown in Figure 5A. HsOrc6-K168A/K169A/D173A and HsOrc6-K169A mutants showed diminished binding with polytene chromosomes as compared to the wild-type HsOrc6 (Figure 5C). Similar to the overexpressed *Drosophila* wild-type Orc6 (37), HsOrc6 did not follow the DNA distribution along chromosomes but preferred less condensed, AT-rich inter-band regions (Figure 5C). Figure 5D shows the expression levels of the wild-type and mutant HsOrc6 proteins isolated from the fly strains used in Figure 5C.

**Figure 6.**
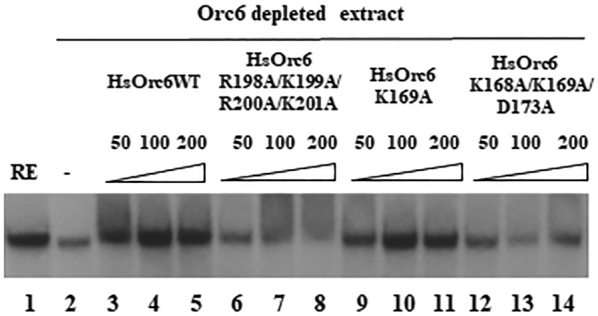
*In vitro* DNA replication in Orc6 depleted *Xenopus* extracts supplemented by the addition of increasing amounts of recombinant wild-type or mutant human Orc6 proteins. *Xenopus* sperm DNA was incubated for 30 min in *Xenopus* extract at a concentration of 2–5 ng/μl in a presence of [α^32^P]dCTP. Where indicated, extracts were depleted for Orc6 by using antibodies raised against human and *Drosophila* Orc6. Add back experiment was performed by the addition of 50, 100 or 200 ng of recombinant human Orc6 proteins to the depleted extracts; RE, non-depleted replication extract control (lane 1). HsOrc6WT (lanes 3–5), HsOrc6-R198A/K199A/R200A/K201A (lanes 6–8), HsOrc6-K169A (lanes 9–11) and HsOrc6-K168A/K169A/D173A (lanes 12–14) were used in rescue experiments. No recombinant protein was added to the Orc6 depleted extract in lane 2.

Using *Xenopus in vitro* DNA replication assay, we have shown that the purified recombinant wild-type HsOrc6 could efficiently restore DNA replication activity in *Xenopus* egg extracts depleted of endogenous Orc6 (42). In these experiments, endogenous *Xenopus* Orc6 protein was depleted from the extracts using antibodies raised against human and *Drosophila* Orc6 proteins (42). Therefore, next, we supplemented Orc6-depleted *Xenopus* extracts with recombinant proteins HsOrc6WT, HsOrc6-R198A/K199A/R200A/K201A, HsOrc6-K169A and HsOrc6-K168A/K169A/D173A to verify their ability to support *in vitro* DNA replication. As expected, the addition of the wild-type HsOrc6 protein restored DNA replication activity of the Orc6 depleted extract (Figure 6, lanes 3–5). However, HsOrc6-R198A/K199A/R200A/K201A and HsOrc6-K168A/K169A/D173A mutant proteins were not able to rescue DNA replication in Orc6 depleted *Xenopus* extract (Figure 6, lanes 6–8 and 12–14). The activity of the human Orc6 with a single Lys169 to Ala mutation (HsOrc6-K169A) was close to the wild-type human Orc6 protein in these experiments (Figure 6).

### The structural model of Orc6/DNA complex

**Figure 7.**
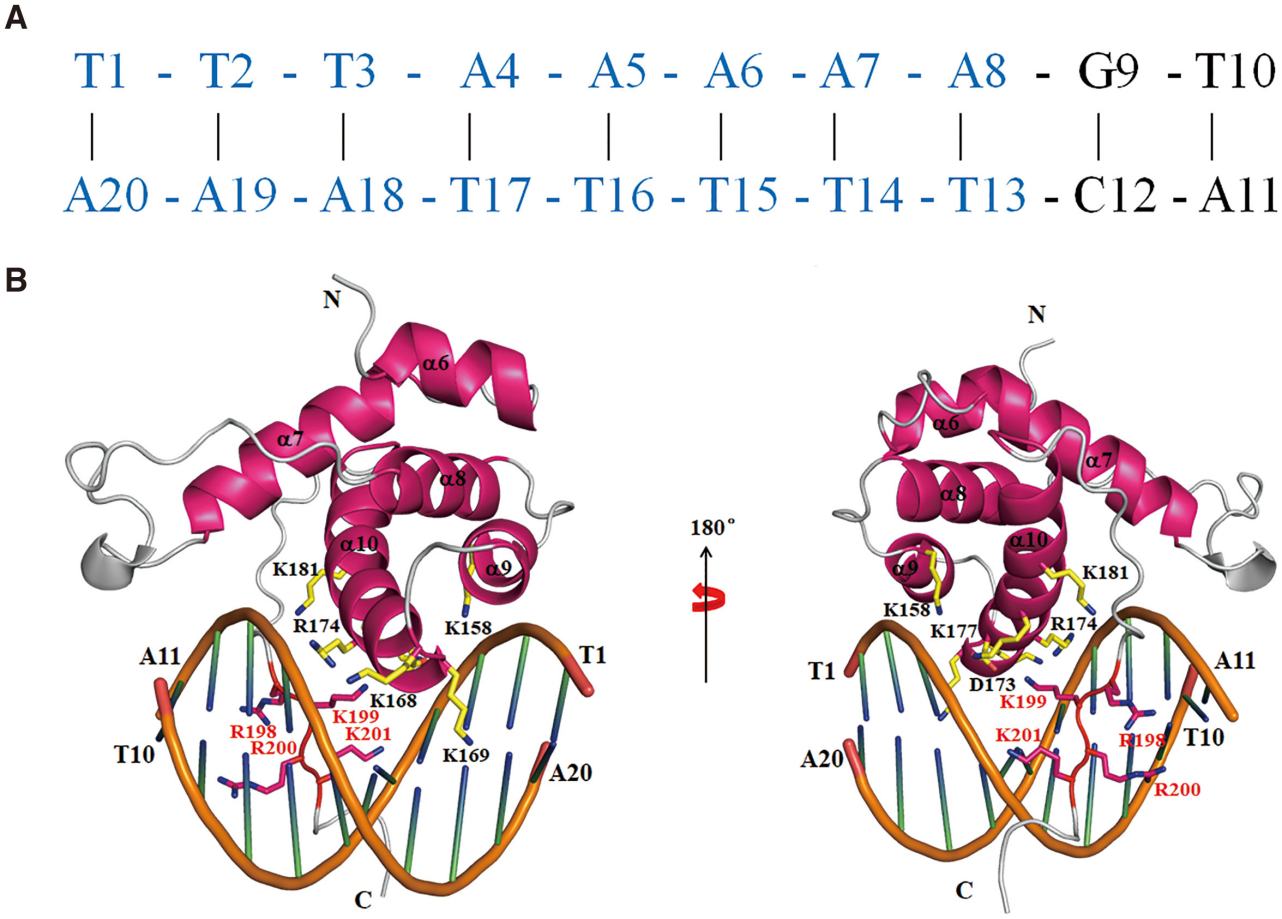
Structure model of Orc6/DNA complex generated by HADDOCK 2.2. (**A**) The sequence of 10 bp DNA used in structural model and the binding sequence with Orc6 is colored by blue. (**B**) Ribbon representation of Orc6/DNA (only residue 95–207 of Orc6 shown) complex in which Orc6 binds DNA like a clamp through the Orc6-M domain and the amino acid cluster, Arg198-Lys199-Arg200-Lys201, of Orc6-C domain.

Using the mapped binding interfaces on DNA and HsOrc6, a structural model for the Orc6/DNA complex was calculated using HADDOCK 2.2 (64) (HADDOCK statistics summarized in Supplementary Table S3). According to the model, HsOrc6 binds DNA like a clamp through the HsOrc6-M and HsOrc6-C domains (Figure 7 and Supplementary Figure S15). The HsOrc6-M domain clamps the major groove containing the T2-A8 and T13-A19 regions of the 10 bp DNA through the interface formed by α9 and α10 helices. Residues Lys168, Lys169, Asp173, Arg174 and Lys177 of Orc6-M domain are inserted into the groove. Residues Lys158, Lys168, Arg174 and K181 have a contact with the sugar phosphate backbone mainly by electrostatic interactions (Figure 7). Interestingly, residues Arg198-Lys199-Arg200-Lys201 of the Orc6-C domain adopt an extended conformation and are inserted into the major groove on the other side. Especially, the extended conformation of Arg198-Lys199-Arg200-Lys201 allows HsOrc6 having a hydrophobic contact with DNA in addition to the charge–charge interaction. The side chains of Arg198 and Lys201 pointing away from each other in combination with Lys199, Arg200 occupy a region covering four A-T base pairs including the A5–A8 and T13-T16 regions (Figure 7). As a result, residues including Lys168, Lys169, Lys177, Arg174 along with Arg198-Lys199-Arg200-Lys201 form a positively charged cleft and act like a clamp to grab the DNA (Supplementary Figure S16), representing a potentially unique mechanism of DNA recognition. Interestingly, no interactions between HsOrc6 and the minor groove of DNA were observed in our model, possibly due to the fact that the minor groove of B'-DNA is narrower than that of B-DNA in our case.

## DISCUSSION

Here, we report the high-resolution NMR structures of full length HsOrc6 comprising of the tandem HsOrc6-N, HsOrc6-M and HsOrc6-C domains. We have also identified the HsOrc6-M domain and the Arg198-Lys199-Arg200-Lys201 amino acid region of HsOrc6-C domain as the DNA binding domains of HsOrc6 termed as HsOrc6-DBD (residues 95-207). Human Orc6 does not have a consensus specific sequence in DNA binding and the required intermolecular NOEs are unavailable for the structure determination of the HsOrc6 in complex with DNA. Therefore, we applied the chemical shift perturbation data obtained from the HsOrc6-DBD titrated with the shortest binding DNA (10bp) to build a structural model of Orc6/DNA complex. The HADDOCK model of the HsOrc6/DNA complex reveals a good complementary fit between HsOrc6 and the groove of DNA suggesting several residues for base-specific interactions that are consistent with the observation in NMR titration experiments (Figure 7). The residues Lys158, Lys168, Lys169, Lys177, Arg174 and Lys181 of α9 and α10 helices along with Arg198-Lys199-Arg200-Lys201 form a positively charged cleft and act like a clamp to grab the DNA (Supplementary Figure S16) representing a potentially unique mechanism of DNA recognition. Although no interactions between HsOrc6 and the minor groove were observed in our HADDOCK model, we cannot rule out the possibility that the residues Arg198-Lys199-Arg200-Lys201 adopt a more extended/twisted conformation resulting in the insertion of Lys/Arg sidechains into the minor groove or that both the HsOrc6-M and the residues Arg198-Lys199-Arg200-Lys201 have contacts with the minor groove of DNA. The displacement of water molecules in the minor groove could also happen when HsOrc6 binds to DNA in solution. Although the electrostatic interactions are observed between HsOrc6 and DNA through the contact with the sugar phosphate backbone, the extended conformation of Arg198-Lys199-Arg200-Lys201 also allows HsOrc6 having a hydrophobic contact with DNA in addition to the charge–charge interaction (Figure 7). This is consistent with our ITC data that indicate that the binding of HsOrc6 to DNA is an endothermic process implying the contribution of hydrophobic interaction (Supplementary Figure S10). Meanwhile, this hydrophobic interaction may also explain the differences among the *K*_D_ values derived from NMR titration data shown in Supplementary Figure S9 (K169 ∼ 47.1 ± 1.4 µM and A170 ∼ 57.7 ± 1.1 µM versus R200 ∼ 9.92 ± 2.91 µM and I203 ∼ 11.3 ± 1.5 µM). Furthermore, our mutation analysis shows that Arg198-Lys199-Arg200-Lys201 and a combination of Lys168-Lys169-Asp173 are the key residues for DNA binding and DNA replication (Figures 5 and 6). Although Lys158 shows a contact with DNA in our model, HsOrc6-K158A does not disrupt the interaction of HsOrc6 with DNA as shown in Supplementary Figure S17. This suggests that K158 may not contribute significantly to DNA binding.

The structure of HsOrc6-M domain has the structural topology typically observed for the TFIIB protein. Searching the DALI database (79) produced hundreds of hits with *z*-scores > 3.5 including structures of the TFIIB protein in the absence and presence of DNA such as human (PDB code:1VOL) and *Pyrococcus woesei* (PDB code:1D3U and 1AIS) TFIIB/DNA complex structure. The structural alignment indicated that HsOrc6-M domain is similar with the C-domain of TFIIB protein in TBP–TFIIB–DNA complex (PDB code: 1D3U) with *z*-score = 5.7. In TBP–TFIIB–DNA complex, the HTH motif of TFIIB protein is inserted into the major groove of DNA. However, in our model the HTH motif (α9-loop-α10) clamps one strand of DNA and the α10 is inserted into the groove of DNA. Specifically, the α9 is more negative than the corresponding helix in TBP–TFIIB–DNA complex that would explain why the α9-loop-α10 can clamp the strand of DNA in our model (Supplementary Figure S18).

Recently, the cryo-EM structure of budding yeast ORC in the complex with DNA was solved (30). This structure included Orc6 protein (residues 271–430) and showed that TFIIB-like domain B of Orc6 (residues 271–386) contacts with the backbone through Tyr277 (30). The structural analysis showed that both HsOrc6-N and HsOrc6-M domains are similar to TFIIB-B domain of yeast Orc6 (Supplementary Figure S19A and B). Interestingly, based on structural analysis it was shown that the loop of the α2-turn-α3 in the HsOrc6-N potentially contacts with DNA through residues such as R41 that gives a large chemical shift change in NMR titration experiments of full-length HsOrc6 with DNA. This suggests the existence of a non-specific charge or a potential cooperative interaction with DNA (Figure 3C and Supplementary Figure S19A), even though the individual HsOrc6-N domain does not appear to bind to DNA based on DNA titration experiment (Supplementary Figure S10A). The further mutation analysis indicates that R41 does not play a major role in the binding with DNA (Supplementary Figure S17). However, the binding region of HsOrc6-M domain we presented here localizes in the opposite direction of the binding sites with DNA compared to the budding yeast ORC suggesting a different role of this motif in the case of human/metazoan Orc6 (Supplementary Figure S19B).

**Figure 8.**
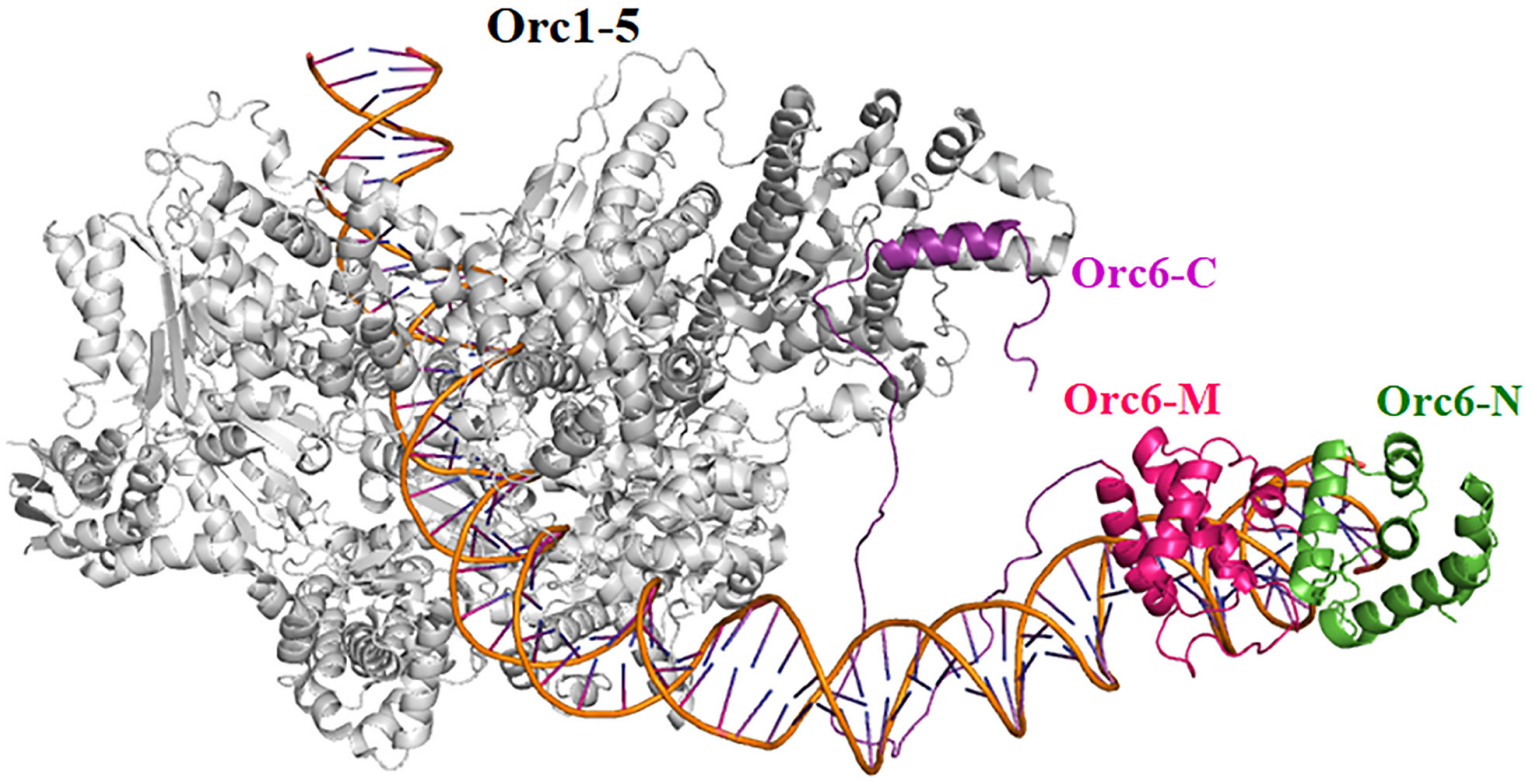
The proposed model of HsORC/DNA complex based on the *Saccharomycescerevisiae* 3 Å cryo-EM structure ORC/DNA complex (PDB code: 5ZR1). The Orc1-5 is shown in gray. The domains of Orc6 are colored as green (Orc6-N), magenta (Orc6-M) and purple (Orc6-C).

In human cells, Orc6 localizes in the nucleus together with other ORC subunits and also associates with chromatin (44,77). HsOrc6, similar to its *Drosophila* homolog, can interact with DNA directly and forms a distinct complex with both human Lamin B2 origin and *Drosophila* ori-β fragments (42). Our data presented here reveal the importance of a highly positively charged motif RKRK (residues 198–202) localized within HsOrc6-C domain just outside of HsOrc6-M for HsOrc6/DNA binding. Interestingly, this motif resembles a nuclear localization signal (NLS) (Supplementary Table S2) (80) and is conserved in humans and rodents but is not present in *Drosophila*. Even though Orc6 is an essential component for the initiation of DNA replication, its functions vary in different eukaryotic species. In yeast *Saccharomycescerevisiae*, Orc6 is an integral part of the ORC complex (30). Moreover, budding yeast Orc6 interacts with Cdt1 protein and facilitates the loading of MCM helicase (81). This function has not yet been confirmed in metazoan species and requires additional studies. *Drosophila* Orc6 is not homologous in sequence to the budding yeast protein, but similarly to the budding yeast Orc6 it is tightly associated with other ORC subunits. In *Drosophila*, Orc6 binds DNA and may participate in positioning of ORC at the origin. HsOrc6, homologous with *Drosophila* protein and required for replication, loosely associates with core ORC subunits, possesses DNA-binding ability and associates with DNA both *in vitro* and *in vivo* (38,42,44,77) (Figures 5,6 and Supplementary Figure S20). Our data suggest an interesting possibility that the presence of an NLS and an ability of HsOrc6 to bind DNA are necessary for the targeting of the ‘loose’ human Orc6 to the nucleus and chromatin ultimately providing an additional anchoring for the ORC association with the DNA. Importantly, as shown in Figure 8, HsOrc6 recognizes DNA through HsOrc6-DBD and may induce a bend in DNA allowing for a tighter binding of the protein. The functional six-subunit ORC complex in human cells is formed after core Orc1-5 joins the initial HsOrc6/DNA complex through the interaction of the α11 helix of HsOrc6-C (aa 231–242) with Orc3, while HsOrc6-N is flanking at this stage (Figure 8). Interestingly, recent time-resolved electron microscopy study showed that the yeast Orc6 N-terminal domain promotes the MCM loading by forming the intermediate MCM–ORC complex via the interaction with MCM2 (82). Although the study of interaction between of HsOrc6 and MCM is beyond the scope of this manuscript, we speculate that HsOrc6-N potentially may play a similar role in human DNA replication (Supplementary Figure S21).

In summary, our results indicate that Orc6 has two structural motifs similar to TFIIB helical domains and suggest the function for Orc6 during DNA recognition and an assembly of larger complexes at the origin regions. According to our model, Orc6 protein possibly acts as a targeting, positioning and an assembly factor to form a functional six-subunit ORC at the origins ultimately resulting in the formation of the pre-RC.

## DATA AVAILABILITY

Atomic coordinates for the reported solution structure have been deposited with the Protein Data bank under accession number 6KVG. Assignments for full-length Orc6 have been deposited in the BioMagResBank (http://www.bmrb.wisc.edu) under accession number 36286.

## Supplementary Material

gkaa751_Supplemental_File
