# Supplementary material for: Structural basis of DNA replication origin recognition by human Orc6 protein binding with DNA
Source: Nucleic Acids Res. 2020 Sep 28;48(19):11146–61. doi: 10.1093/nar/gkaa751 (PMC7641730; doi:10.1093/nar/gkaa751)
Supplement: gkaa751_Supplemental_File [file gkaa751_supplemental_file.pdf]

Supplementary Information

Structural Basis of DNA Replication Origin Recognition by Human Orc6 Protein Binding with DNA

Naining Xu[1,2†], Yingying You[1,3†], Changdong Liu[1†], Maxim Balasov[4†], Lee Tung Lun[1], Yanyan Geng[1], Chun Po Fung[1], Haitao Miao[1], Honglei Tian[1], To To Choy[1], Xiao Shi[1], Zhuming Fan[5], Bo Zhou[1], Katarina Akhmetova[4], Hongyu Yang[2], Quan Hao[5], Igor Chesnokov[4*], and Guang Zhu[1, 6*]

[1] Division of Life Science, The Hong Kong University of Science and Technology, Clear Water Bay, Kowloon, Hong Kong SAR, China
[2] Department of Oral and Maxillofacial Surgery, Peking University Shenzhen Hospital, Guangdong, China
[3] Department of Otolaryngology, Xiangya Hospital, Central South University, Changsha, Hunan, China
[4] Department of Biochemistry and Molecular Genetics, University of Alabama at Birmingham School of Medicine, Birmingham, United States
[5] School of Biomedical Sciences, University of Hong Kong, 21 Sassoon Road, Hong Kong
[6] State Key Laboratory of Molecular Neuroscience, The Hong Kong University of Science and Technology, Clear Water Bay, Kowloon, Hong Kong SAR, China

* To whom correspondence should be addressed. Email: ichesnokov@uab.edu and gzhu@ust.hk

[†] The authors wish it to be known that, in their opinion, the first 4 authors should be regarded as joint First Authors.

Figure. S1 Sequence alignment of Orc6 from different species: Q9Y5N6, *H. sapiens*; Q2HJF3, *Bos taurus*; Q9WUJ8 *Mouse*; Q9Y1B2, *D. melanogaster*; P38826, *S. cerevisiae*; O74796, *S. pombe*. Sequences were taken from the UniProt database (www.uniprot.org). The secondary structure of HsOrc6 is depicted above the alignment. Sequences were aligned with ClustalW2(1) and analyzed with Jalview 2 (2).

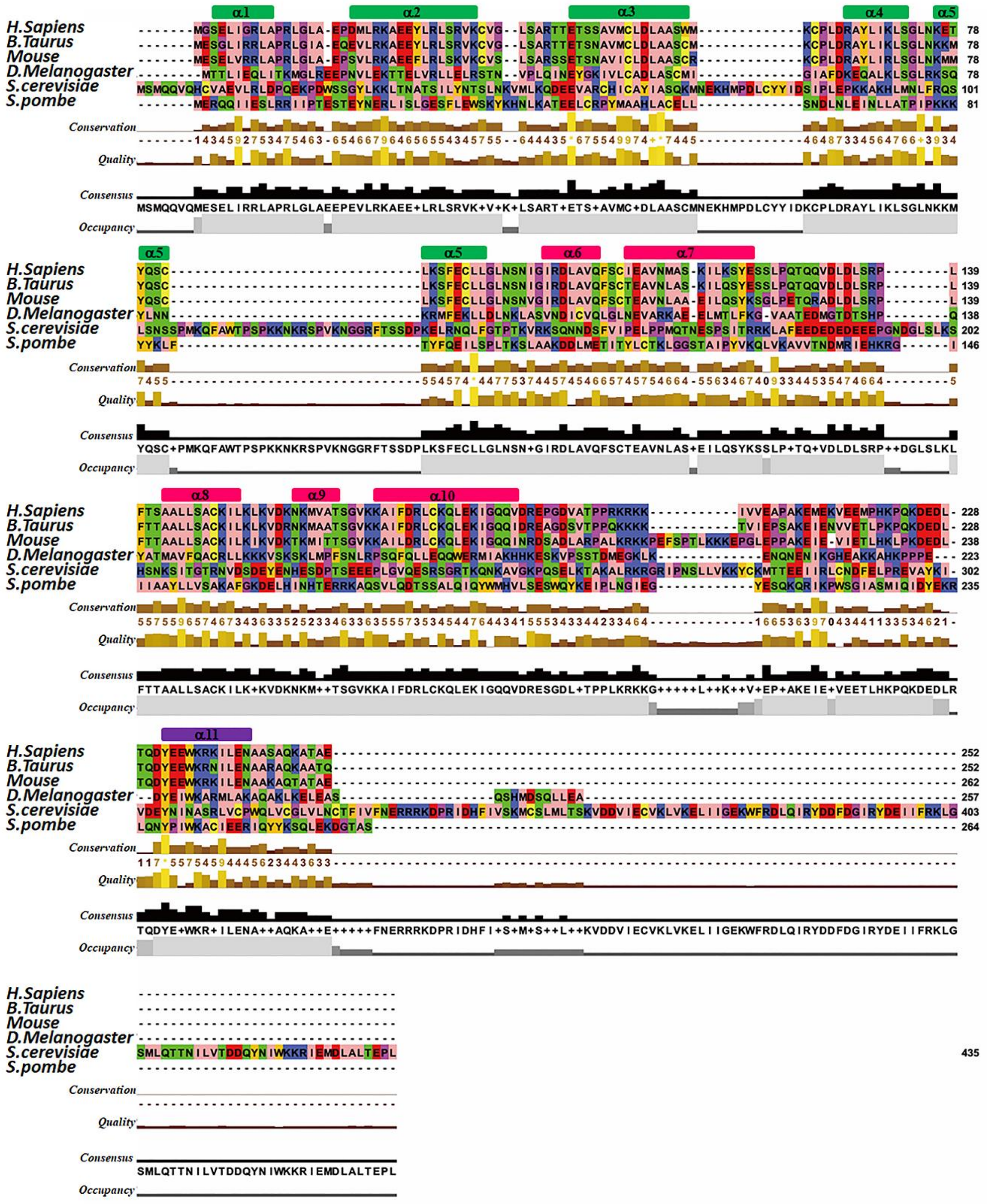

Figure. S2 (A) Differences in chemical shifts of individual HsOrc6 domains with full length HsOrc6 are plotted versus residue number. (B) Differences in chemical shifts of the HsOrc6 construct containing residues 1-207 with full length HsOrc6 are plotted versus residue number.

A

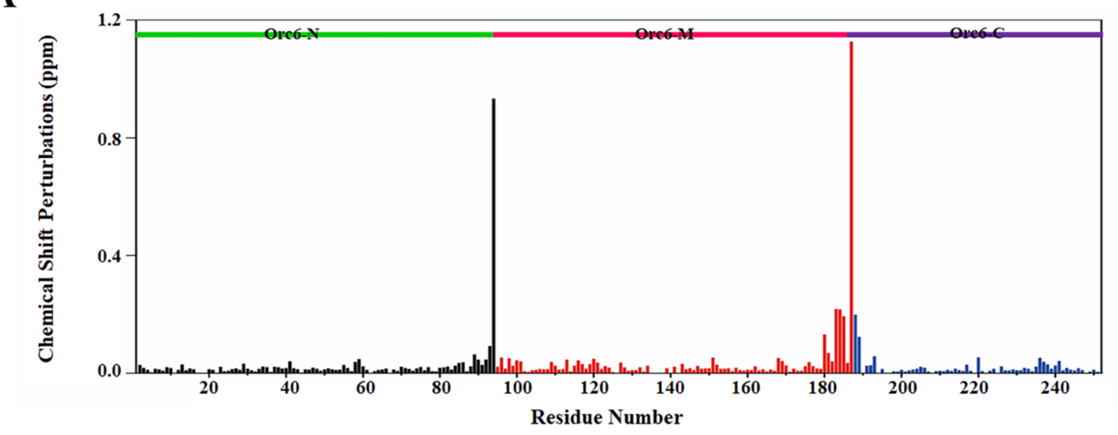

B

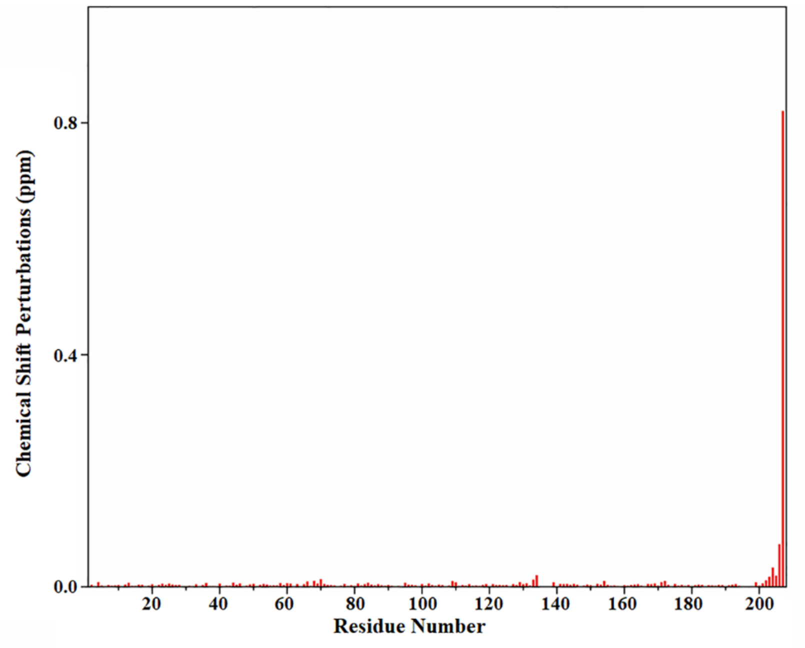

Figure. S3 The $^{1}H,^{15}N$ HSQC NMR spectra of (A) HsOrc6-N (residue 1-94) titrated with HsOrc6-M (residue 95-187) and (B) HsOrc6-N+M (residue 1-187) titrated with Orc6-C (residue 188-252). Spectra are colored as: free protein (red), with ratio 1:2 (green) and with ration 1:5 (blue).

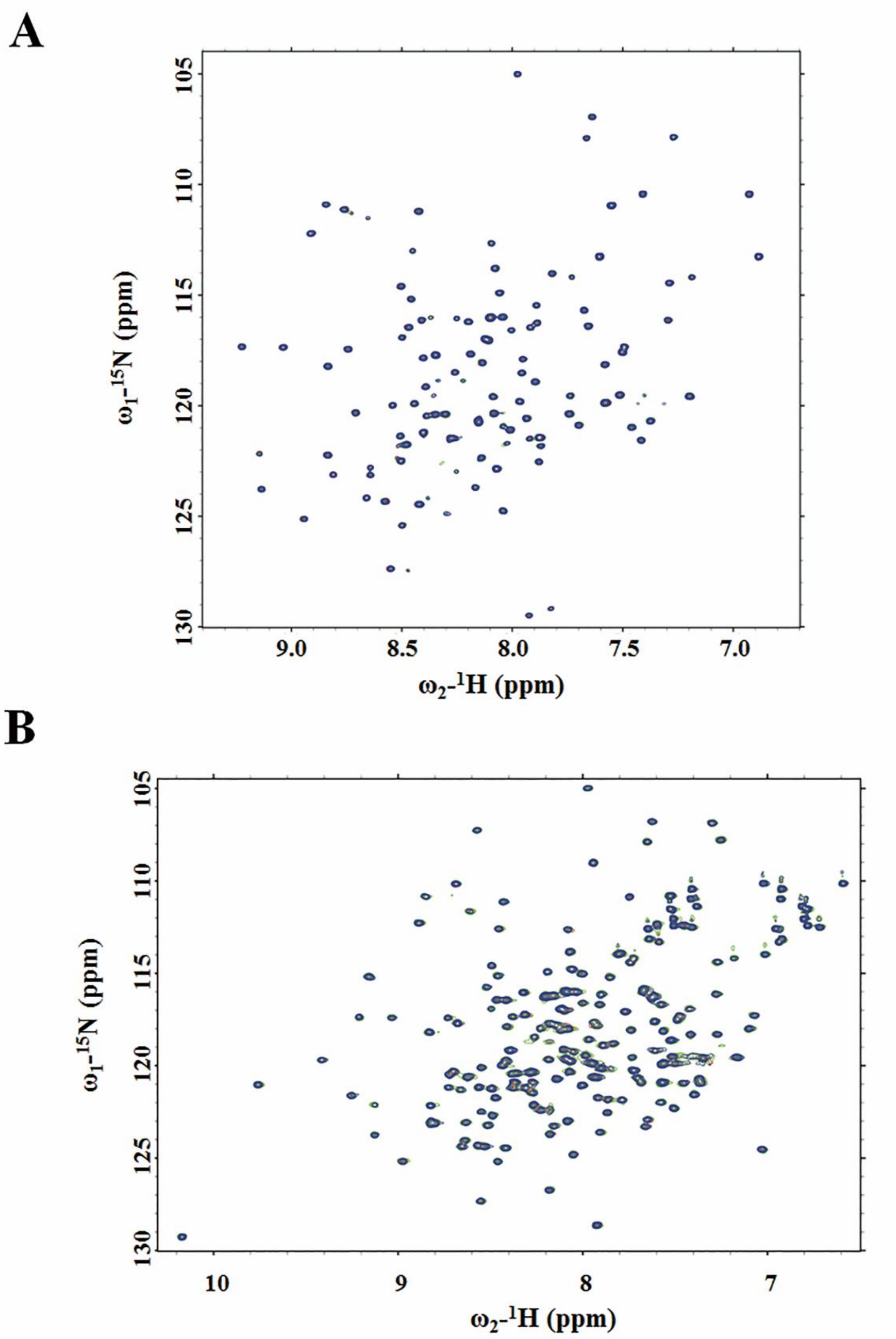

Figure. S4 The $^{1}H,^{15}N$ HSQC NMR spectrum of full length HsOrc6 with assignment.

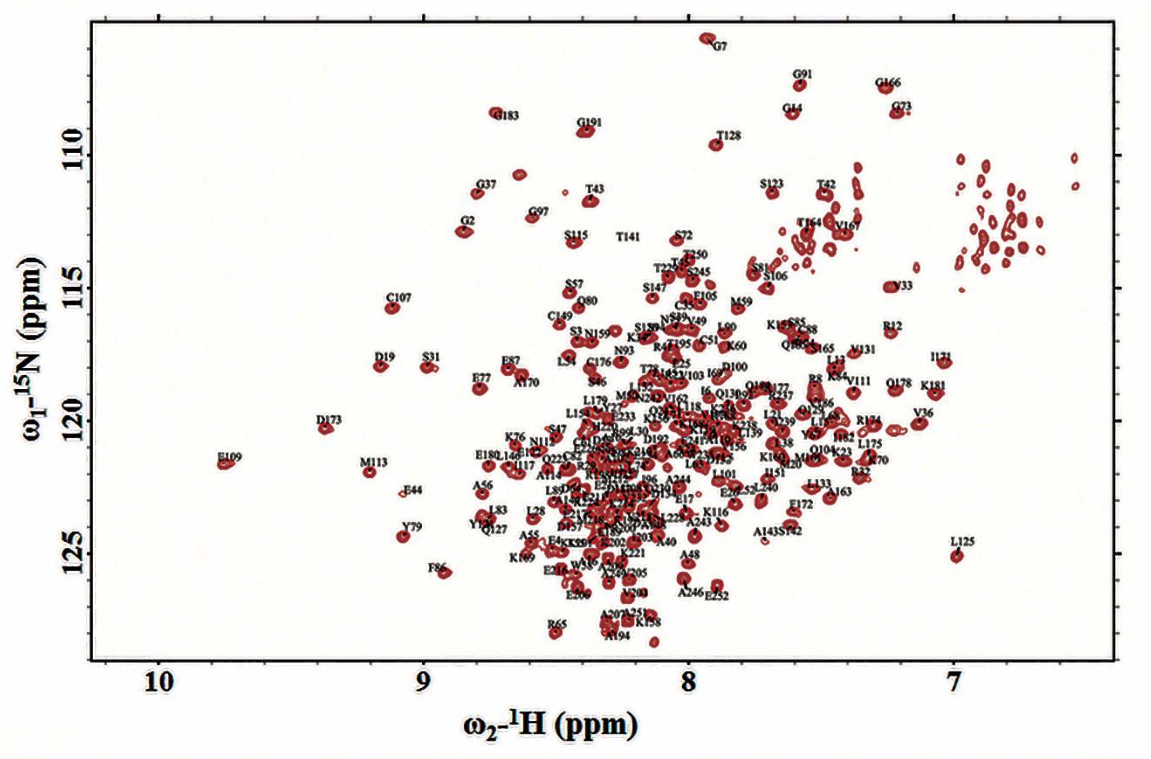

Figure. S5 {^{1}H}-^{15}N heteronuclear NOE values of full length HsOrc6 in apo form measured at 298 K and pH 6.5.

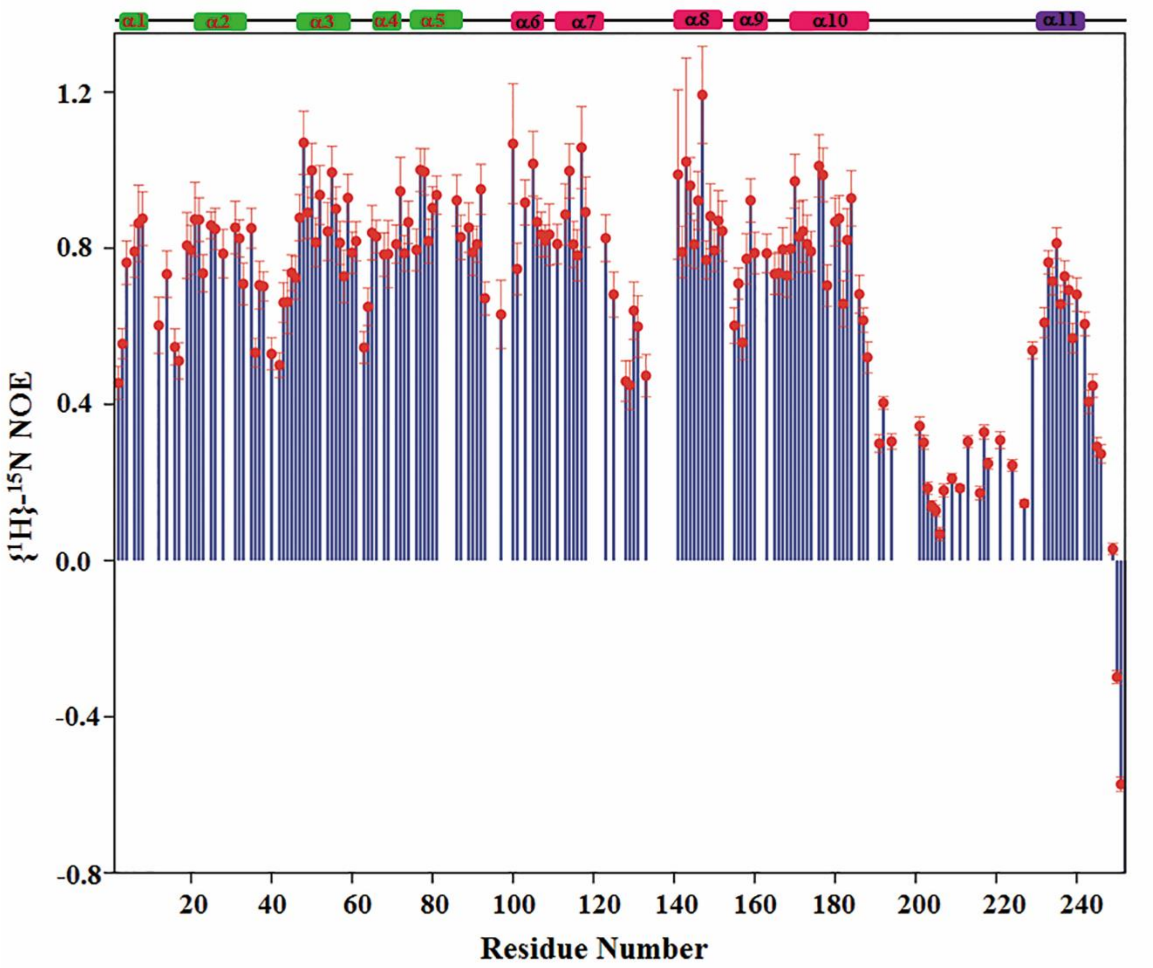

Figure. S6 The structures of the hydrophobic cage of HsOrc6-N (A) and HsOrc6-M (B) are shown with the side chains of important hydrophobic residues indicated in yellow. (C)The structural superimposition of HsOrc6-N (green) to HsOrc6-M (magenta). The angular difference between α3 in HsOrc6-N and α8 in HsOrc6-M is ~20°. (D) The structural superimposition of HsOrc6-M (magenta) to the reported crystal HsOrc6-M structure (blue) (PDB code: 3M03). The formed helix located in the linker between α7 and α8 containing residues Gln127-Leu133 is indicated by a red dashed circle.

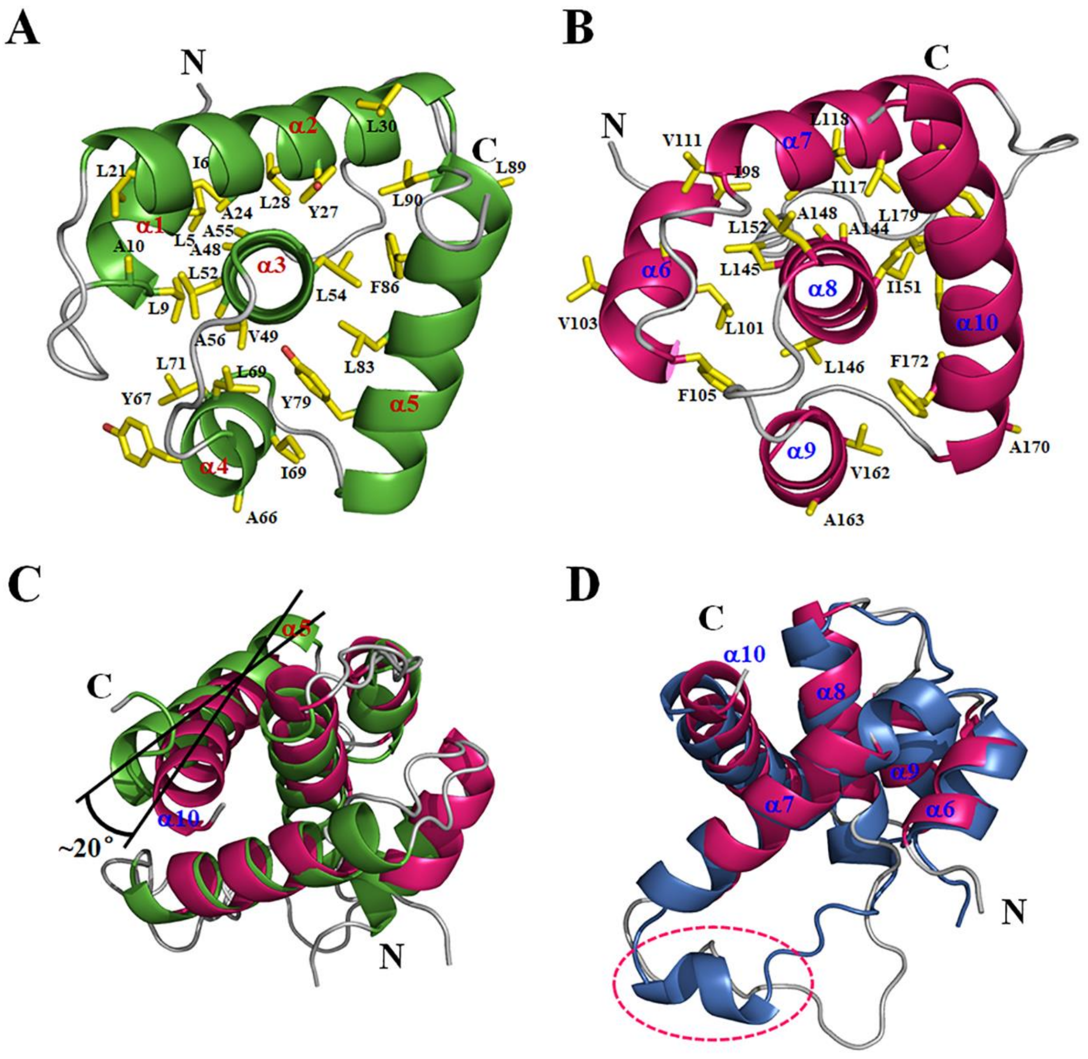

Figure. S7 Titration curves for representative HN resonances of full length HsOrc6 binding with 17bp DNA. The ^{1}H,^{15}N combined chemical shift perturbations δΔ (ppm) ($\Delta\delta = [(\Delta\delta HN)^2+(\Delta\delta N/5)^2]^{0.5}$) were plotted as a function of the DNA:HsOrc6 molar ratios. The dissociation constants (K_D) were obtained by fitting the experimental data as described in Material and Methods.

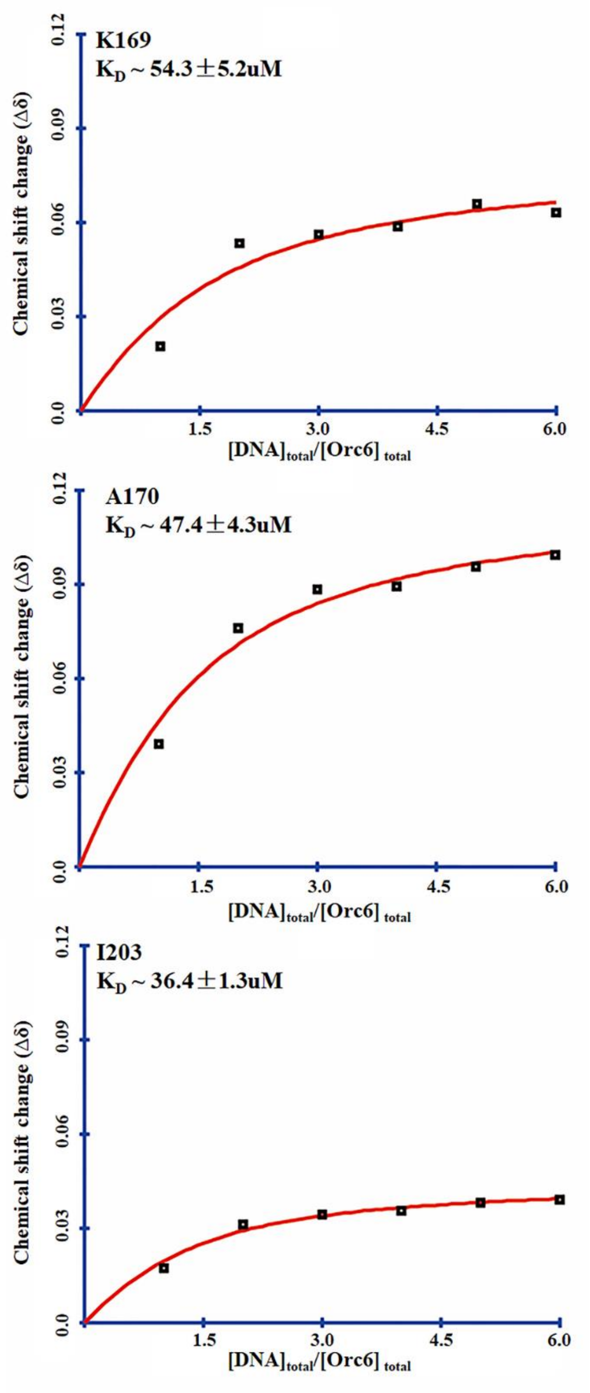

Figure. S8 The overlaid $^1H,^{15}N$-HSQC spectra of ^{15}N-labeled HsOrc6-N (residue 1-94) (A), ^{15}N-labeled HsOrc6-M (residue 95-187) (B) and ^{15}N-labeled HsOrc6-C (residue 188-252) (C) in free form (red) and titrated with 17b DNA at a molar ratio of 1:1 (green), 1:2 (purple) and 1:5 (blue). Residues that undergo significant changes in chemical shifts upon formation of the complex with DNA are highlighted by arrows and labeled with peak assignments.

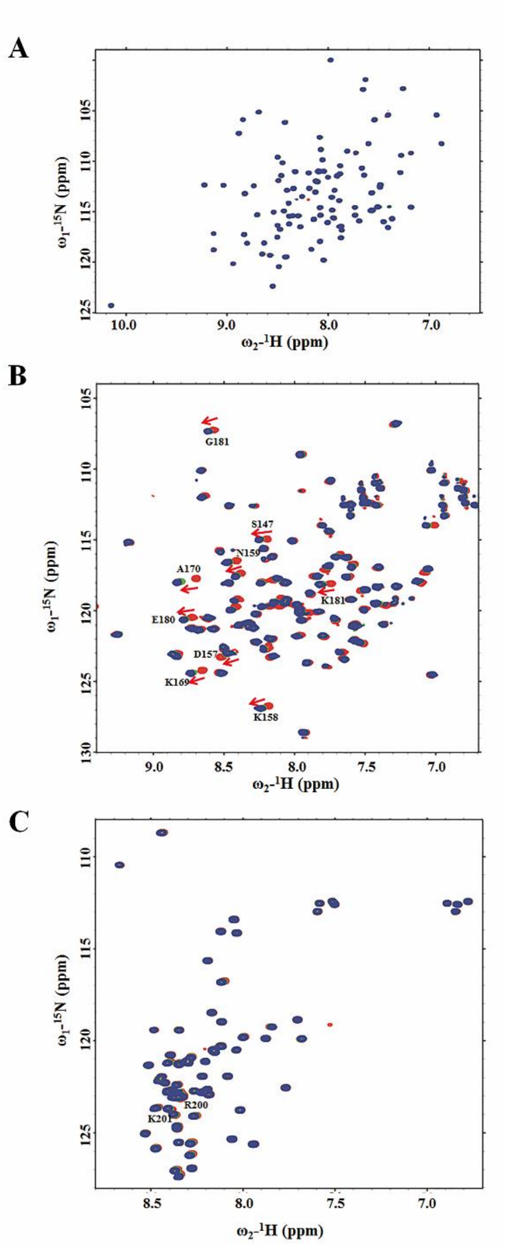

Figure. S9 (A) The overlaid $^1H,^{15}N$-HSQC spectra of ^{15}N-labeled HsOrc6-DBD (residue 95-207) in free form (red) and titrated with 17b DNA at a molar ratio of 1:1 (green), 1:2 (purple) and 1:5 (blue). Residues that undergo significant changes in chemical shifts upon formation of the complex with DNA are highlighted by arrows and labeled with peak assignments. (B) Weighted chemical shift perturbations for backbone ^{15}N and ^1HN resonances as calculated by the equation $\Delta\delta = [(\Delta\delta HN)^2+(\Delta\delta N/5)^2]^{0.5}$. The two solid lines indicate the mean $\Delta\delta$ value and the mean $\Delta\delta$ value plus 1 SD. (C) Titration curves for representative HN resonances of HsOrc6-DBD binding with 17bp DNA. The $^1H,^{15}N$ combined chemical shift perturbations $\delta\Delta$ (ppm) were plotted as a function of the DNA: HsOrc6-DBD molar ratios. The dissociation constants (K_D) were obtained by fitting the experimental data as described in Material and Methods.

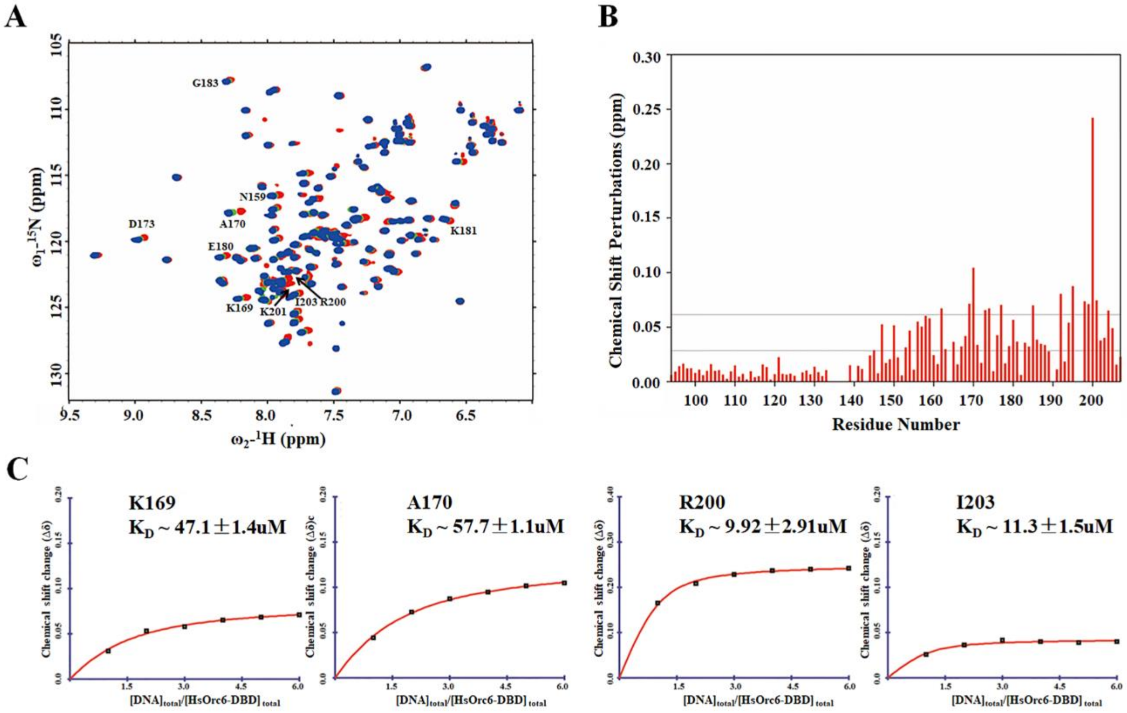

Figure. S10 Isothermal titration calorimetry showing (A) full length HsOrc6 interaction with 17bp AT-rich DNA, (B) full length HsOrc6 interaction with 17bp GC-rich DNA, (C) HsOrc6-N interaction with 17bp AT-rich DNA and (D) HsOrc6-4A mutant interaction with 17bp AT-rich DNA. Top panels: enthalpic heat absorbed versus time at 25°C during titrations. Bottom panels: thermogram of the integrated peak intensities plotted against the molar ratio of the complex. Best-fit curves using single-site binding model. The DNA sequence: 17-bp AT-rich (5′-GGCCCTTTTTTTTCTAG-3′ and 5′-CTAGAAAAAAAAGGGCC-3′); 17-bp GC-rich (5′-GCGTCCGGCCCGCTGCC-3′ and 5′-GGCAGCGGGCCGGACGC-3′).

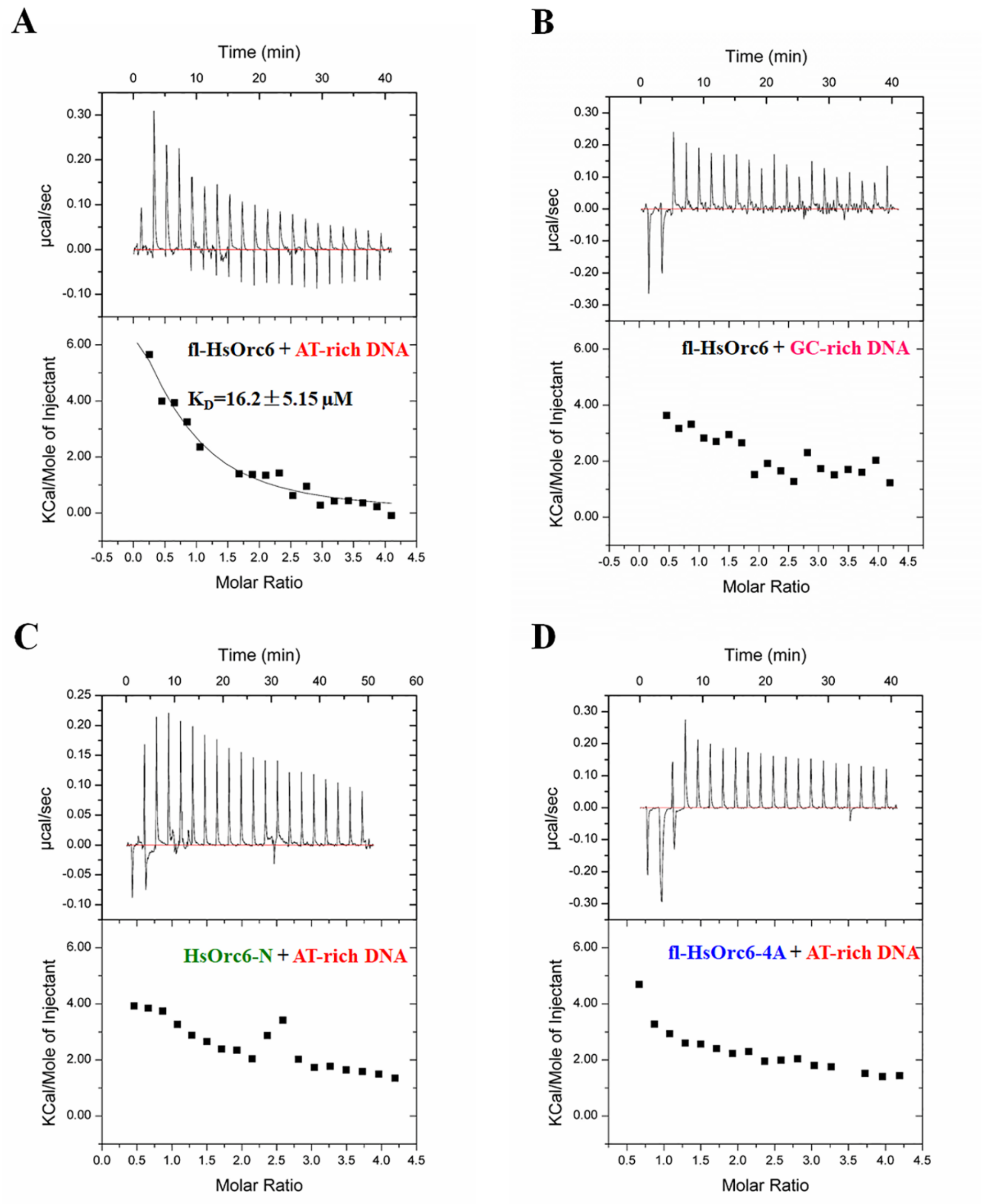

Figure. S11 Superposition of $^{1}H,^{15}N$-HSQC spectra of HsOrc6-DBD (residue 95-207) with titrations of different lengths of DNA (9bp,10bp, 11bp and 12bp). (A) and (B) represent two regions of the spectrum with free protein indicated in red, a molar ration of 1:2 indicated in green, and a molar ratio of 1:5 indicated in blue.

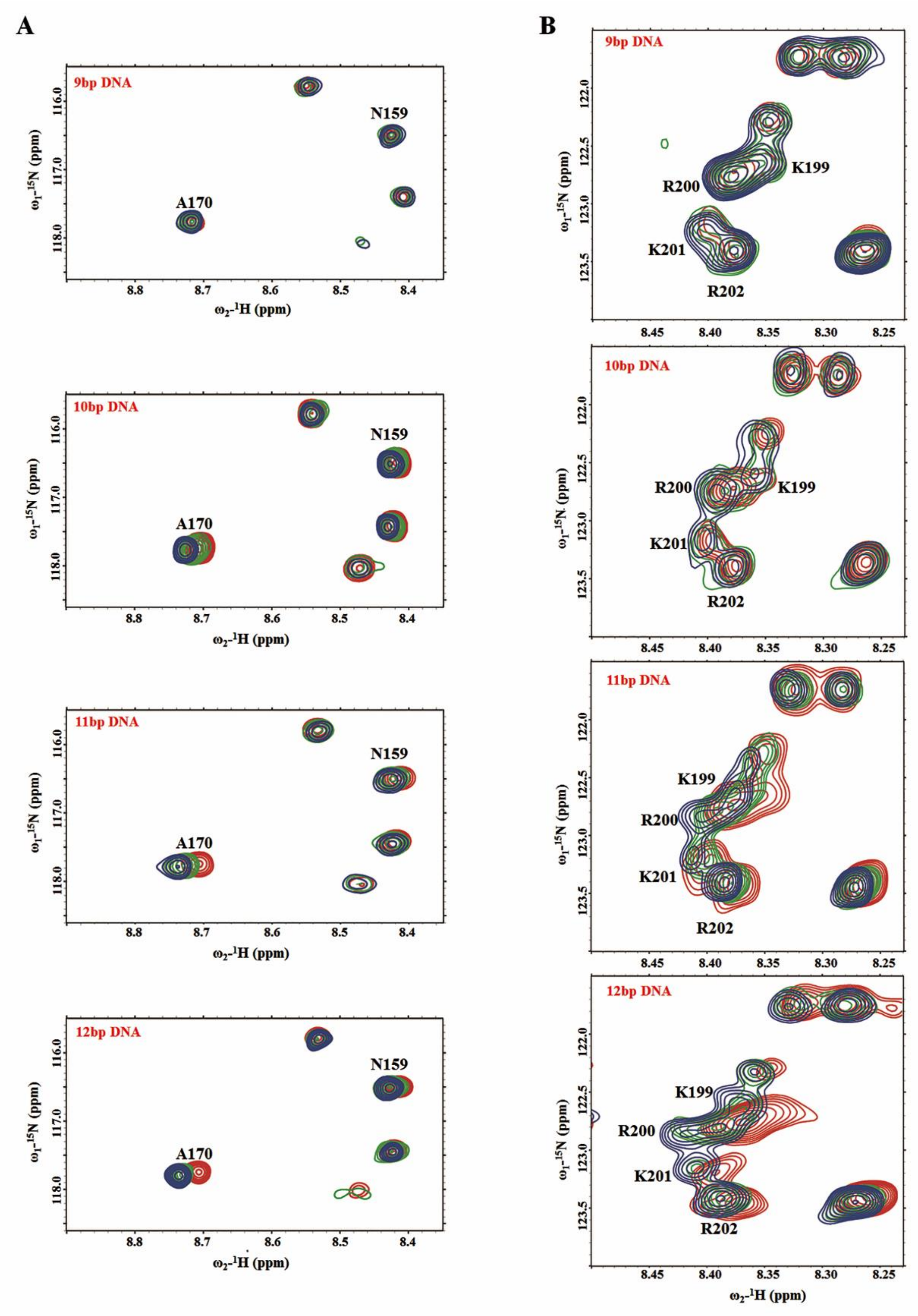

Figure. S12 Titration curves for representative HN resonances of HsOrc6-DBD (residues 95-207) binding with AT-rich DNA of different lengths. The ^{1}H,^{15}N combined chemical shift perturbations δΔ (ppm) ($\Delta\delta = [(\Delta\delta HN)^2+(\Delta\delta N/5)^2]^{0.5}$) corresponding to spectra shown in Figure S11 were plotted as a function of the HsOrc6-DBD:DNA molar ratios. The dissociation constants (K_D) were obtained by fitting the experimental data as described in Material and Methods.

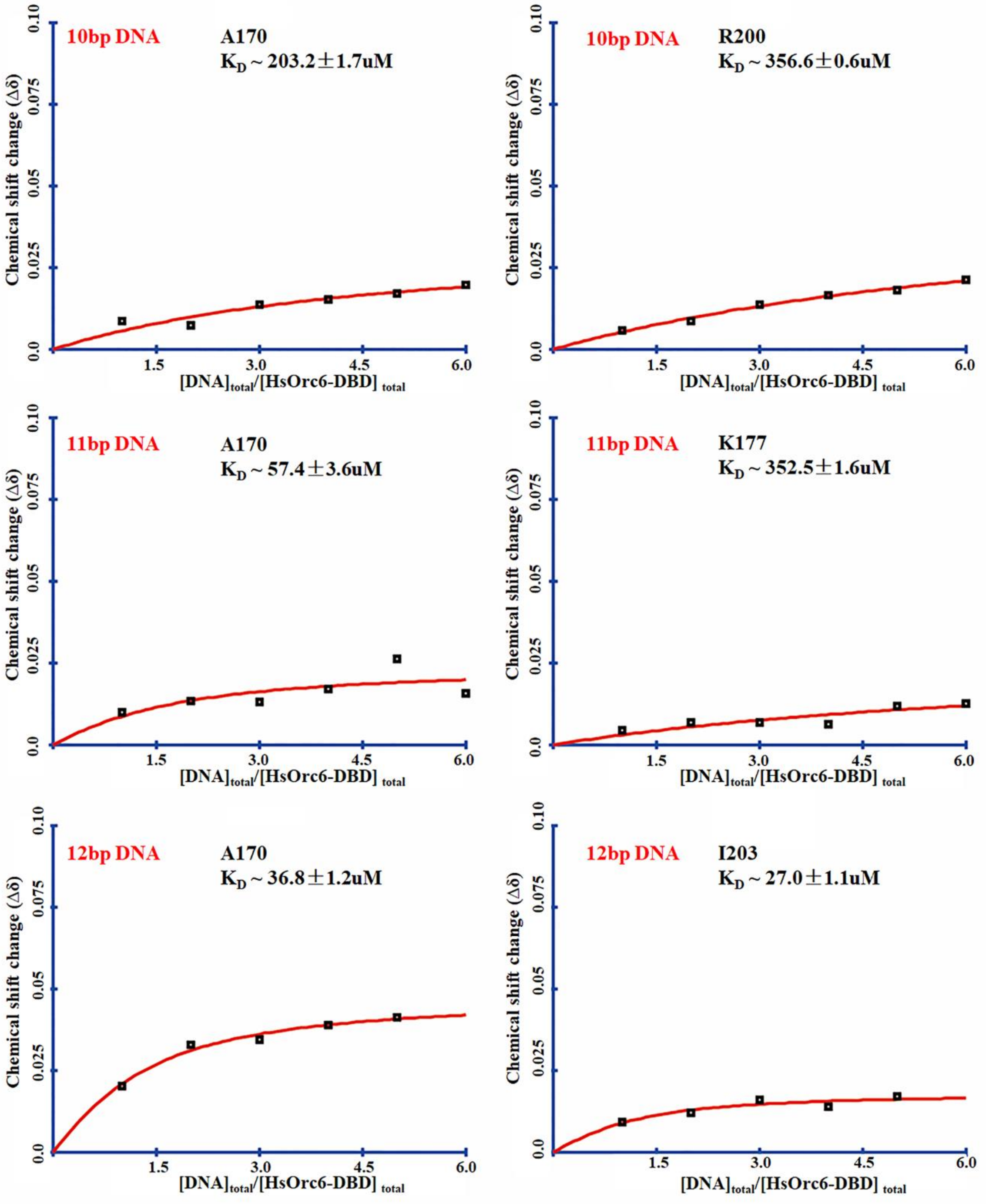

Figure. S13 Relaxation data of free and 17bp DNA bound HsOrc6-DBD (residues 95-207). (A) R_1, (B) R_2, (C) R_2 / R_1, (D) {^{1}H}-^{15}N heteronuclear NOE and (E) τ_c values of free (black) and bound form (red) measured at 298 K on 500 MHz NMR spectrometer are shown. In (E), τ_c values were estimated using the residues in helical segments of HsOrc6-DBD.

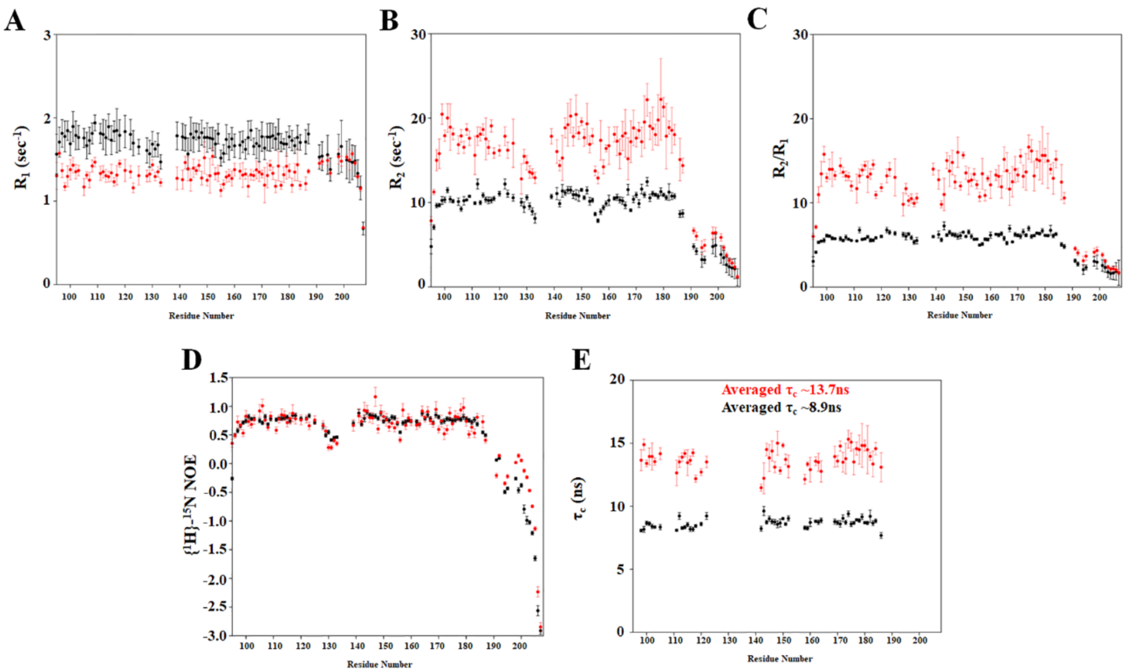

Figure. S14 The ^{1}H,^{15}N HSQC NMR spectra of (A) HsOrc6-3A, (B) HsOrc6-4A, (C) HsOrc6-R41A and (D) HsOrc6-K158A.

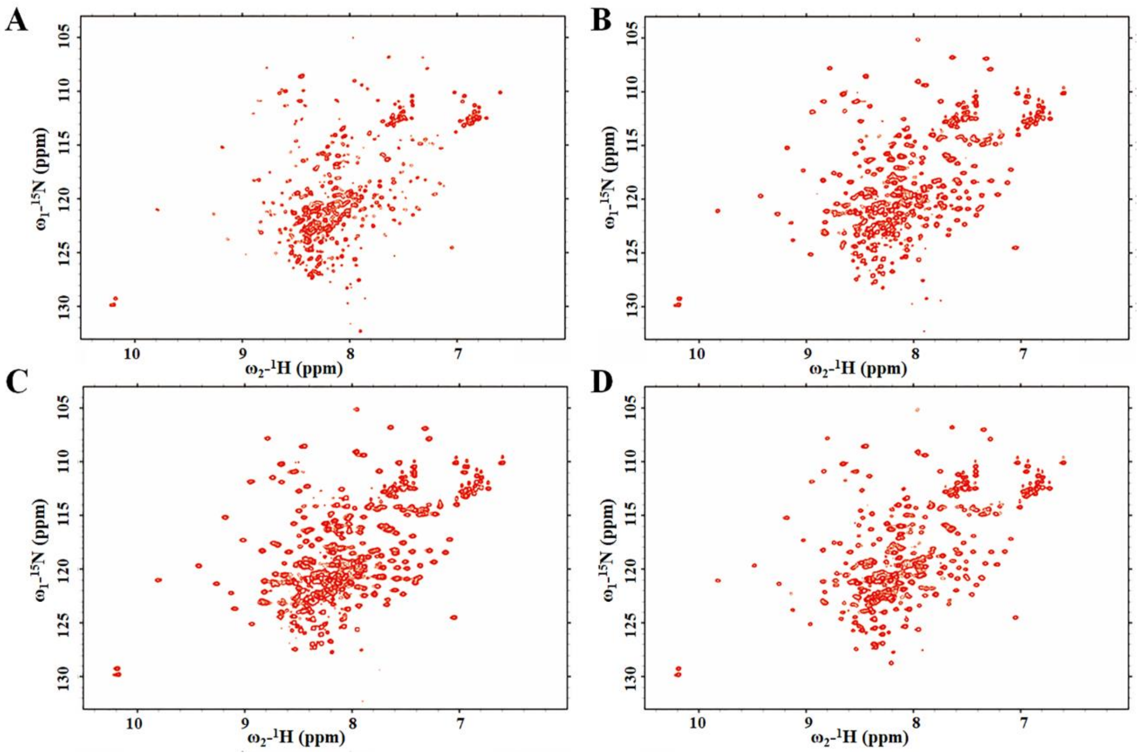

Figure. S15 The HADDCOK model of full length HsOrc6/DNA complex. The secondary structure of each domain is colored as green in HsOrc6-N, magenta in HsOrc6-M and in purple for HsOrc6-C, while the loops are colored gray. The region containing Arg198-Lys199-Arg200-Lys201 is shown in red.

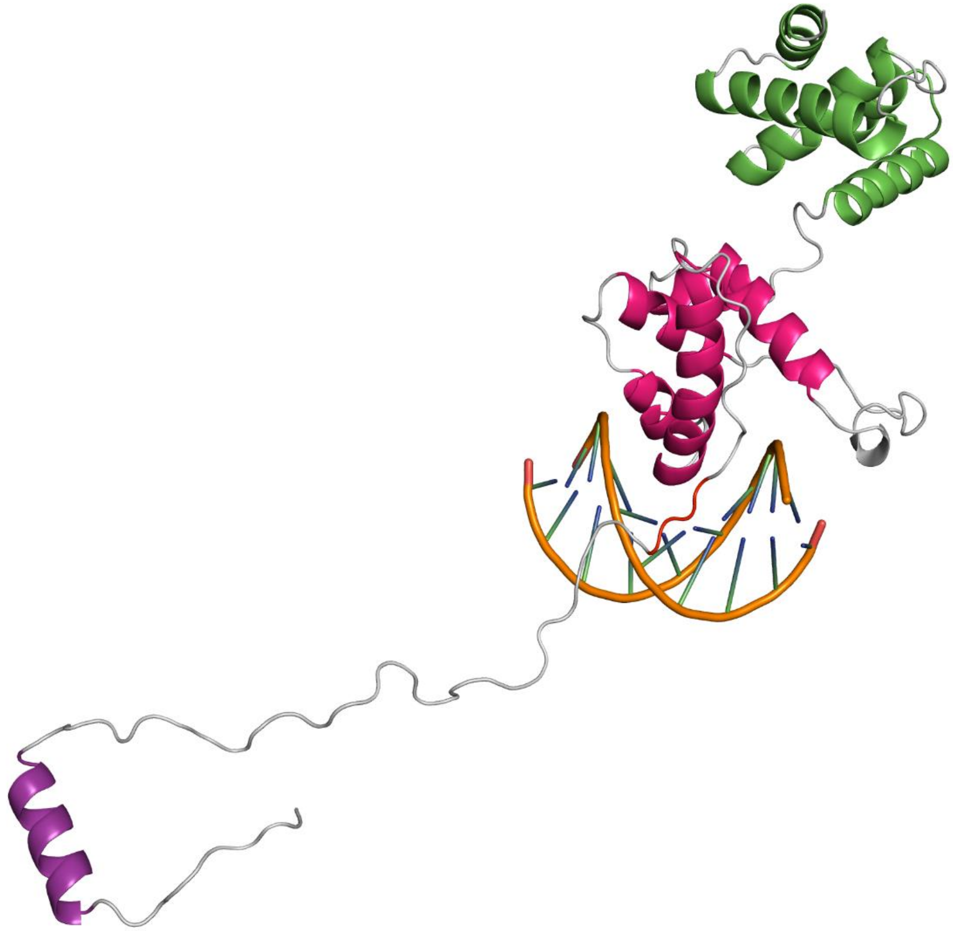

Figure. S16 The electrostatic surface potential of HsOrc6-DBD (residues 95-207) in complex with DNA. The red and blue colors in surface representations denote negative and positive charges, respectively.

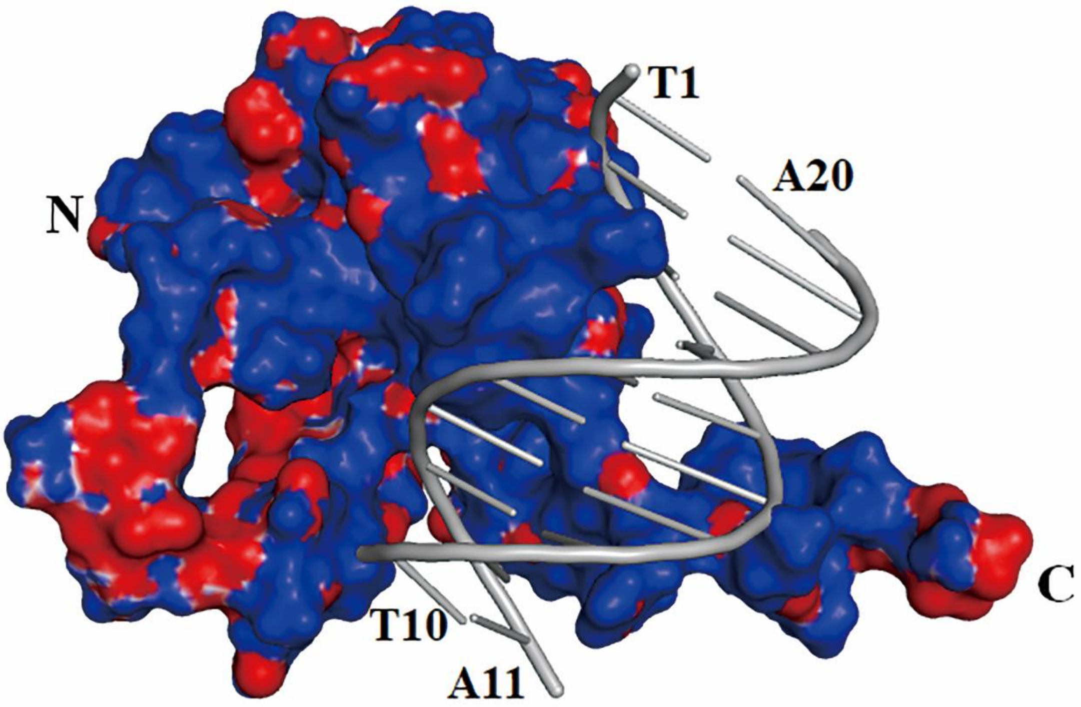

Figure. S17 Overlaid ^{1}H,^{15}N-HSQC spectra of (A) ^{15}N-labeled full length HsOrc6- R41A and (B) ^{15}N-labeled full length HsOrc6-K158A in free form (black) titrated with 17b DNA at a molar ratio of ~ 1:3 (red).

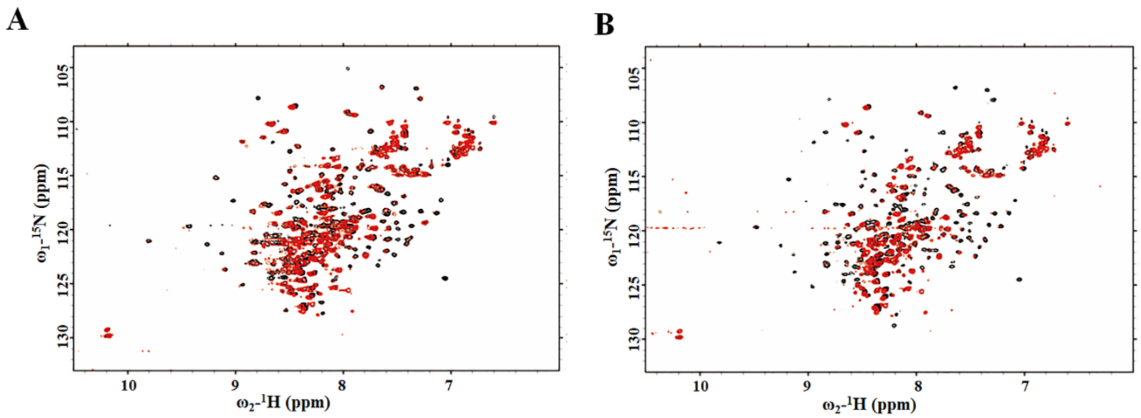

Figure. S18 (A) Structure comparison of HsOrc6-DBD (residues 94-207) (gray) with the C- domain of transcription factor TFIIB (magenta) (PDB code:1D3U). (B) Electrostatic surfaces of the C- domain of TFIIB (PDB:1D3U). The corresponding α9 of HsOrc6-DBD (residues 95-207) is indicated by dashed circles. The red and blue colors in surface representations denote negative and positive charges, respectively.

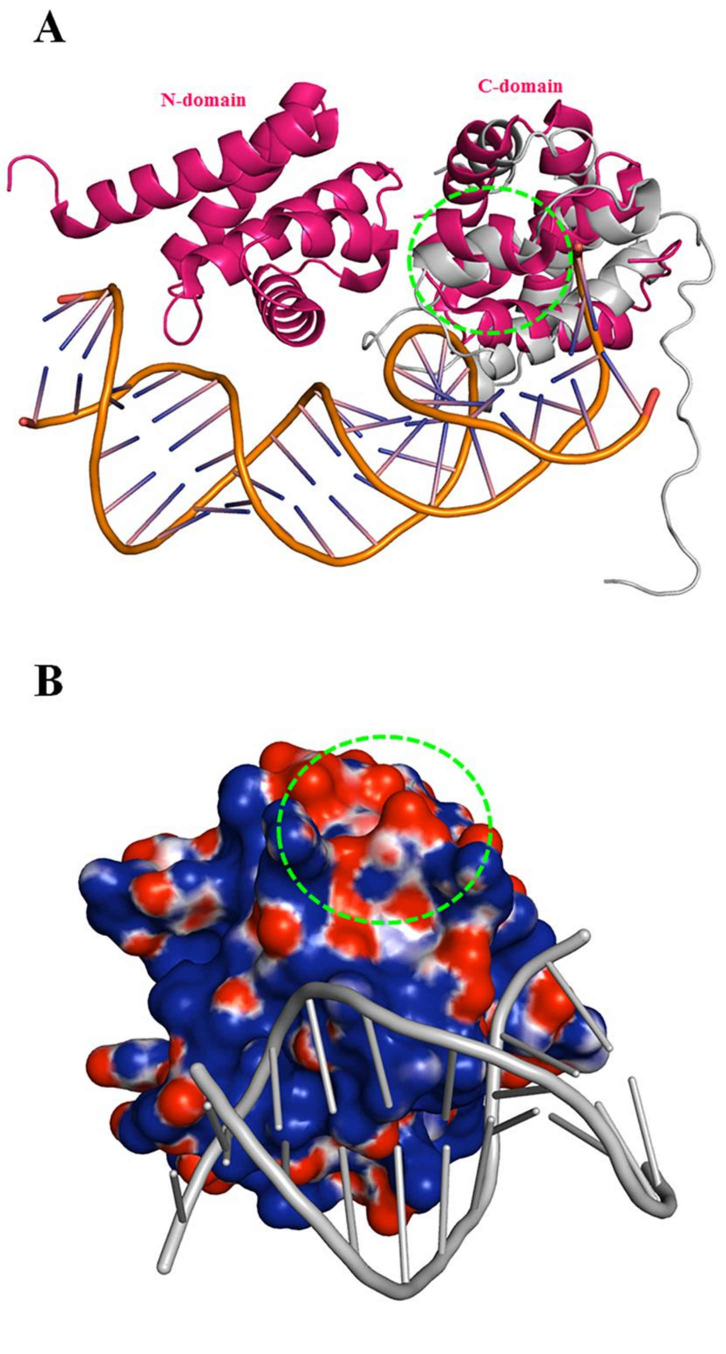

Figure. S19 Structure comparison of (A) HsOrc6-N (residues 1-94, green) and (B) HsOrc6-M (residues 95-187, magenta) with the yeast Orc6 (blue), the transcription factor TFIIB domain B (residues 271–386), in Cryo-EM structure of yeast ORC/DNA complex (PDB code:5ZR1) respectively. The residue Y277 of yeast Orc6 which interacts with DNA and the residue R41 of HsOrc6-N are shown in stick respectively in (A). The region of HsOrc6-M binding with DNA is shown in the red circle in (B).

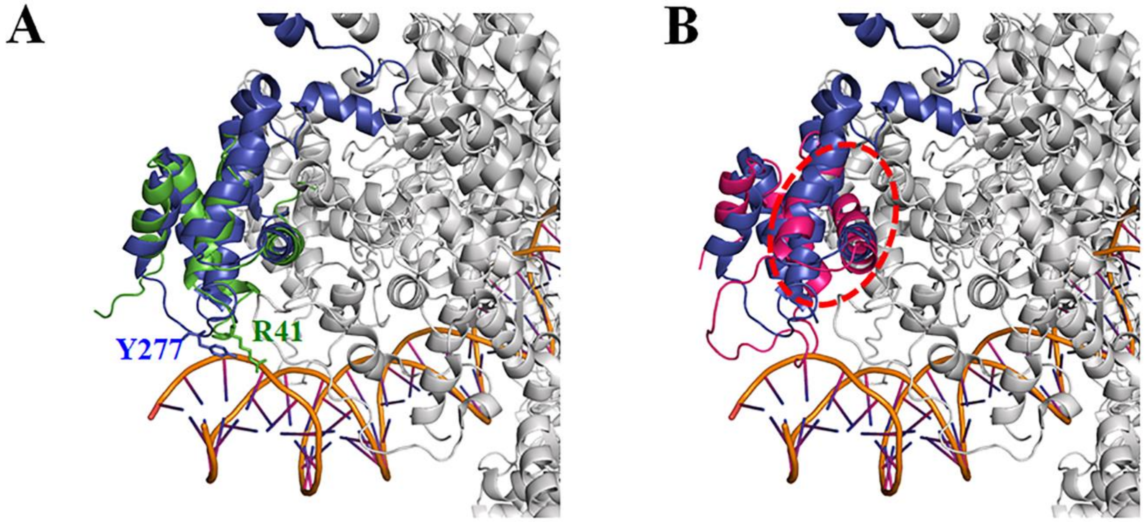

Figure. S20 Salivary glands polytene chromosomes were prepared as described in Materials and Methods and immunostained with mouse monoclonal *Drosophila* Orc5 and rabbit polyclonal human (A) or *Drosophila* Orc6 (B) antibodies. Molecular Probes goat anti-mouse Alexa fluor 568 and goat anti-rabbit Alexa Fluor 488 were used as a secondary antibodies to visualize Orc5 (red) and Orc6 (green) simultaneously. Three representative co-localization sites are indicated.

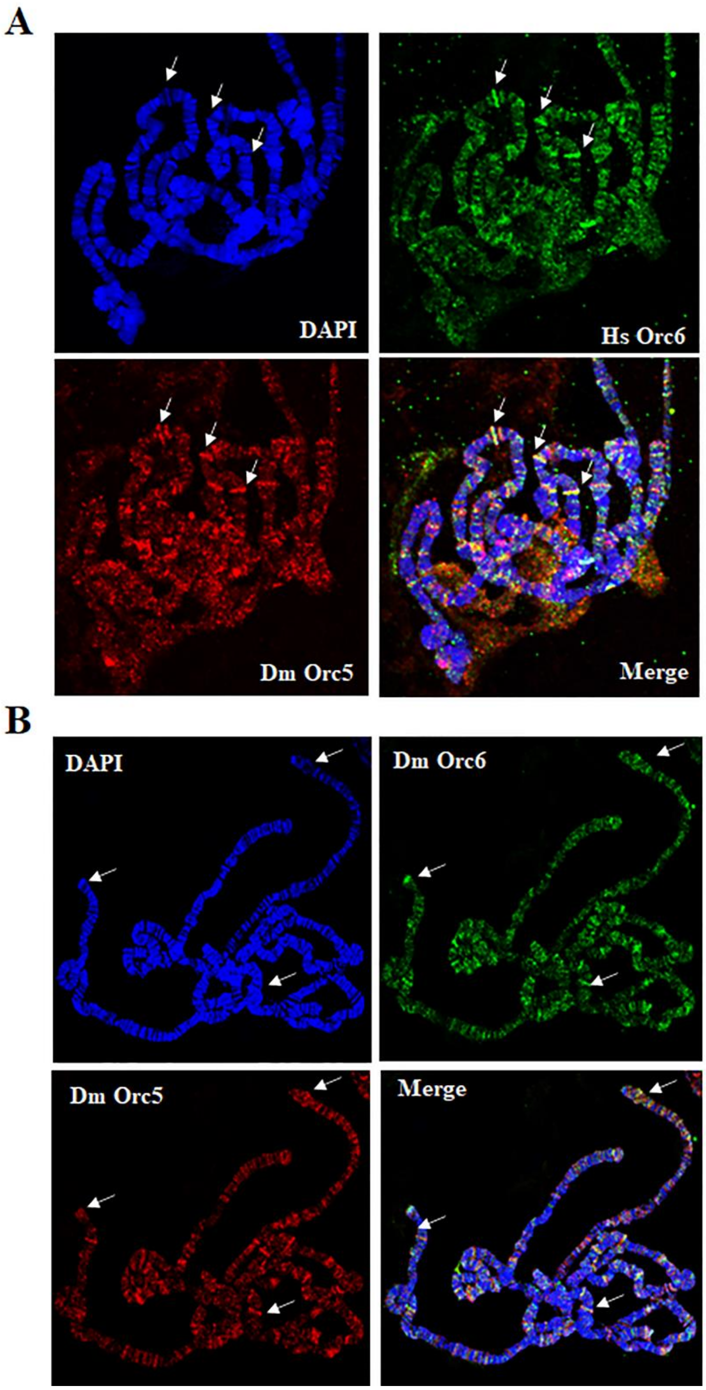

Figure. S21 The proposed model of an MCM loading intermediate, HsORC/MCM/DNA complex, based on the *S. cerevisiae* 4.4 Å cryo-EM structure ORC/MCM/DNA complex (PDB code:6RQC). The Orc1-5 and MCM are shown in cyan and gray, respectively. The domains of Orc6 are colored as green (Orc6-N), magenta (Orc6-M) and purple (Orc6-C).

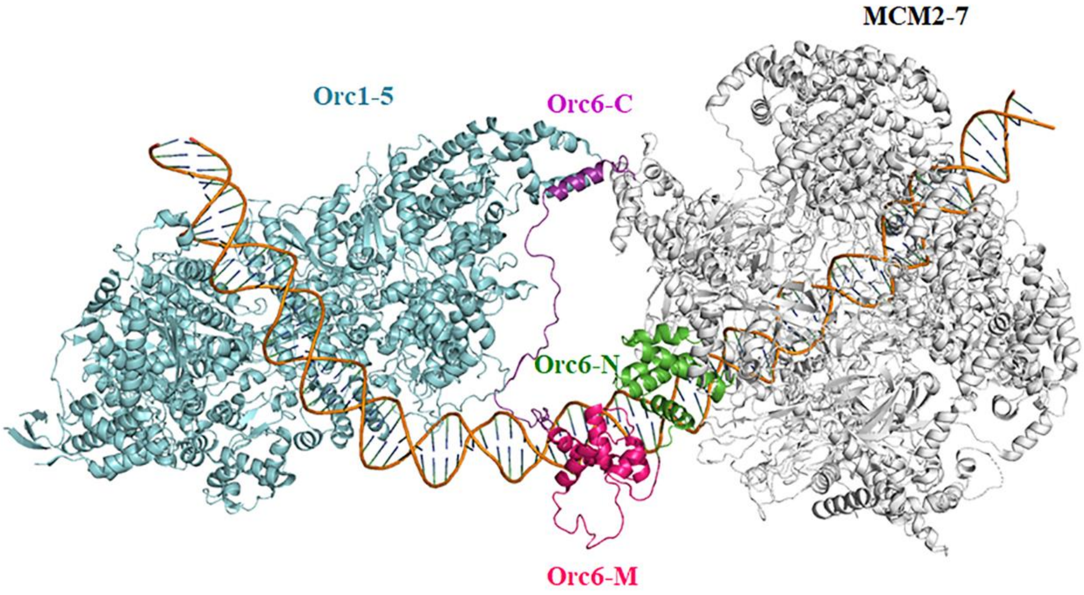

Table S1. Structural statistics for full length HsOrc6

(a) NMR restraints		
Total Experimental Restraints		***3804***
Total NOE Distance Restraints		***3345***
	Short-range, |i-j|<=1	***806***
	Medium-range, 1<|i-j|<5	***1034***
	Long-range, |i-j|>=5	***1505***
	Hydrogen bond	***165***
Dihedral angle restraints		
	Phi	***147***
	Psi	***147***
(b) Violations		
	Number of NOE violations > 0.5Å	***0 ± 0***
	R.m.s. deviation (Å) from experimental distance restraints	***0.0100 ± 0.0001***
	Number of dihedral angle constraint violations > 5°	***0 ± 0***
	R.m.s. deviation (°) from experimental torsion restraints	***0.2575 ± 0.0607***
(c) Deviations from idealized geometry		
	Bonds (Å)	***0.00133 ± 0.0008***
	Angles (°)	***0.30771 ± 0.0063***
	Improper (°)	***0.16535 ± 0.01437***
(d) Structural RMSD to the mean coordinate		
	region (residue number)	***bb/heavy (Å)***
	3-10,19-34,44-59,65-72,76-90;1-94	***0.46/1.18; 0.88/1.54***
	98-105,111-123,142-152,158-165,169-186;95-187	***0.51 /1.17; 1.96/2.75***
	232-242;188-252	***0.30/2.1; 17.15/17.59***
(e) Ramachandran plot (% residues)		
	Residues in most favored regions	***81.80%***
	Residues in additional allowed regions	***14.00%***
	Residues in generously allowed regions	***3.40%***
	Residues in disallowed regions	***0.80%***

Table S2. The comparison of known nuclear localization signal (NLS) sequences including the simian virus 40 large T-antigen (SV40 T-tag), nucleoplasmin and the oncoprotein c-Myc with HsOrc6 DNA binding motif. K and R residues are in red; PAA and LD sequences are in green and blue, respectively(3).

Signal source	Signal sequence
SV40 T-tag	PKKKRKV
Nucleoplasmin	KR-*10amino acid*-KKKK
c-Myc	PAAKRVKLD
HsOrc6	KRKK

Table S3. HADDOCK statistics evaluated on the water refined models of full length HsOrc6 in complex with DNA. The calculations were performed classifying the amino acids experiencing significant chemical changes during titration as interacting residues. The reported data are related to the best 10 structural models of the main clusters with the lowest HADDOCK-scores.

HADDOCK score	-146.6 +/- 25.4
Van der Waals energy	-1068.6 +/- 19.8
Electrostatic energy	-10647.4 +/- 61.3
Desolvation energy	22.1 +/- 2.1
Buried Surface Area	2521.2 +/- 186.0
RMSD from average structure on interface	2.6 +/- 1.6
Restraints violation energy	18.3 +/- 3.8

1. Larkin, M.A., Blackshields, G., Brown, N.P., Chenna, R., McGettigan, P.A., McWilliam, H., Valentin, F., Wallace, I.M., Wilm, A., Lopez, R. *et al.* (2007) Clustal W and Clustal X version 2.0. *Bioinformatics*, **23**, 2947-2948.
2. Waterhouse, A.M., Procter, J.B., Martin, D.M., Clamp, M. and Barton, G.J. (2009) Jalview Version 2--a multiple sequence alignment editor and analysis workbench. *Bioinformatics*, **25**, 1189-1191.
3. Makkerh, J.P., Dingwall, C. and Laskey, R.A. (1996) Comparative mutagenesis of nuclear localization signals reveals the importance of neutral and acidic amino acids. *Curr Biol*, **6**, 1025-1027.
